# The Modular Organization of Protein Interactions in *Escherichia coli*

**DOI:** 10.1371/journal.pcbi.1000523

**Published:** 2009-10-02

**Authors:** José M. Peregrín-Alvarez, Xuejian Xiong, Chong Su, John Parkinson

**Affiliations:** 1 Program in Molecular Structure and Function, Hospital for Sick Children, Toronto, Ontario, Canada; 2 Department of Molecular Biology and Biochemistry, University of Malaga, Malaga, Spain; 3 Department of Biochemistry, University of Toronto, Toronto, Ontario, Canada; 4 Department of Molecular Genetics, University of Toronto, Toronto, Ontario, Canada; Tel-Aviv University, Israel

## Abstract

*Escherichia coli* serves as an excellent model for the study of fundamental cellular processes such as metabolism, signalling and gene expression. Understanding the function and organization of proteins within these processes is an important step towards a ‘systems’ view of *E. coli*. Integrating experimental and computational interaction data, we present a reliable network of 3,989 functional interactions between 1,941 *E. coli* proteins (∼45% of its proteome). These were combined with a recently generated set of 3,888 high-quality physical interactions between 918 proteins and clustered to reveal 316 discrete modules. In addition to known protein complexes (e.g., RNA and DNA polymerases), we identified modules that represent biochemical pathways (e.g., nitrate regulation and cell wall biosynthesis) as well as batteries of functionally and evolutionarily related processes. To aid the interpretation of modular relationships, several case examples are presented, including both well characterized and novel biochemical systems. Together these data provide a global view of the modular organization of the *E. coli* proteome and yield unique insights into structural and evolutionary relationships in bacterial networks.

## Author Summary

Genes and their protein products do not operate in isolation, but form components of highly interconnected biological systems. Identifying the connections between components is therefore critical to understanding how these processes are organized and operate. *E. coli* is the leading model bacterium; however despite its importance in biological and medical discovery, an accurate atlas of these interactions is still lacking. On the other hand, several computational and experimental procedures have been applied on a high-throughput basis to provide collections of interaction data of varying quality and coverage. Using a sophisticated mathematical framework, we have combined and benchmarked these data to create a single, highly reliable set of interactions that encompasses almost 50% of the *E. coli* proteome. Organizing these data on the basis of their interactions, we identify groups of proteins representing functionally coordinated modules such as molecular machines (e.g., the flagellum) and biochemical pathways. Finally through examining the organization of *E. coli* interactions in the context of evolution, we propose a new model of bacterial network evolution that accounts for the integration of foreign genes acquired through horizontal gene transfer mechanisms.

## Introduction

*Escherichia coli* is the leading model bacterium. Due to its ease of culture and genetic manipulation, it has proven extremely useful for the study of basic biological processes including signalling, metabolism and gene expression [1],[2]. Furthermore, *E. coli* serves as a major model for the study of bacterial pathogenesis [3]. In consequence, a considerable body of knowledge has been collated for *E. coli*. First sequenced in 1996, over half of its genes have now been experimentally characterized [2],[4]. In addition, through decades of painstaking biochemical studies a variety of metabolic, signalling and regulatory pathways have been assembled [2],[5],[6]. However, despite the impressive nature of the available data, details of the organization and co-ordination of proteins within and across cellular processes in *E. coli* is still far from complete, precluding a global ‘systems’ view of the *E. coli* proteome.

To date a variety of methods have been developed and systematically applied to derive large scale networks of protein-protein interactions (PPIs) for a variety of organisms. These range from the experimental: e.g. co-immunoprecipitation (co-IP), yeast-two-hybrid (Y2H) screens and tandem affinity purification (TAP) coupled with mass spectrometry [7]–[12]; to the theoretical: e.g. genome context methods and co-expression data [13]–[15]. Exploiting the topological properties of these networks, clustering algorithms have subsequently allowed proteins to be organized into functional modules such as protein complexes or signalling pathways [11],[16]. Integration of additional datasets such as comparative and functional genomics data are further providing insights into how these modules and their components are co-ordinated or how they may have evolved [11],[17]. For example, clustering of phylogenetic profiles in the context of metabolic networks have identified evolutionary conserved functional entities [18].

While a number of genome scale protein-protein interaction (PPI) datasets have been generated for yeast [7],[8],[10],[11],[19],[20], similar datasets for *E. coli* are more modest. These include two datasets of physical interactions generated through TAP [9],[21] and several datasets of functional interactions derived through genome context methods, gene co-expression analyses and literature surveys [13]–[15],[22]. Note that throughout, we use the term *functional* interactions, to represent proteins that may be involved in a common biological process but do not necessarily physically interact. A recurring challenge in the analysis of PPI datasets has been the discrimination of physiologically meaningful interactions (true positives) from those that arise as methodological artefacts (false positives) [23],[24]. To address this challenge integrative methods, such as the use of Bayesian classifiers, have been applied to identify those interactions which are more reliable [20],[25],[26]. More recently three large scale PPI datasets have been generated for *E. coli* based mainly on genome context methods [15],[27],[28]. While these datasets provide extensive coverage, such coverage may compromise the quality of interactions.

Here we build on these previous studies by integrating several experimental and computational interaction datasets to reconstruct an extensive network of functional interactions for *E coli* with an equivalent accuracy to that obtained for small scale (e.g. co-IP) experiments. We combine this set of functional interactions with a recently generated set of physical interactions generated through reciprocal TAP [27] to yield a single global network of over 7,600 high quality protein interactions representing over half of the proteins in *E. coli*. Through the application of a graph clustering algorithm we systematically organize these data into discrete functional modules to provide, to the best of our knowledge, the first large scale view of the modular organization of a bacterial (as opposed to eukaryotic) proteome. Due to the fundamental role of *E. coli* in basic and biomedical research, the findings presented in this study are expected to find significant and wide scale impact.

## Results/Discussion

### A high quality network of functional interactions for *E. coli*

**Figure 1:**
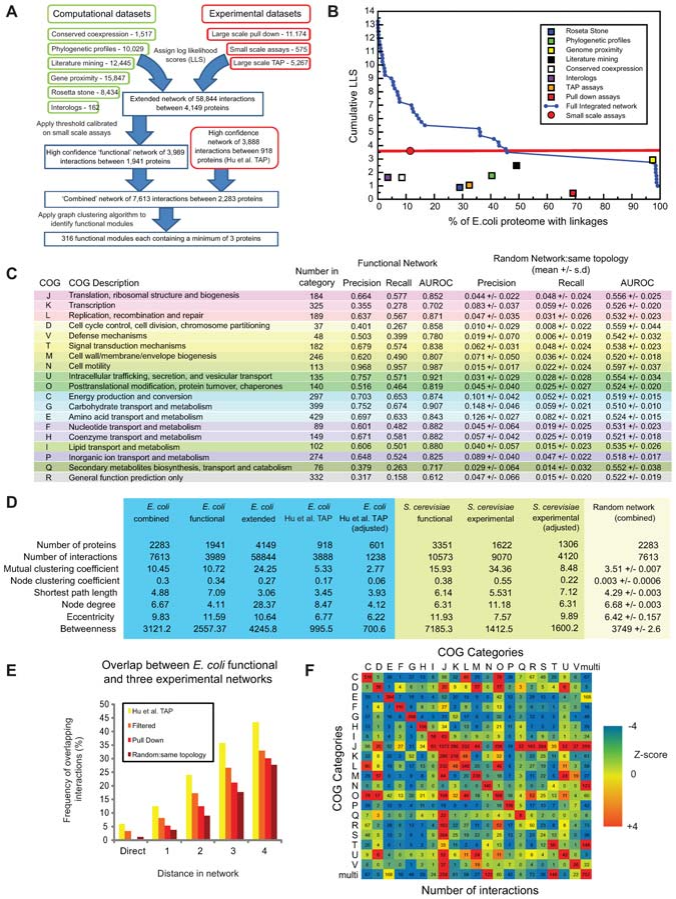
Generation of the global *E. coli* functional network. A: Schematic overview of the generation of the *E. coli* functional network, its integration with the *Hu et al. TAP* dataset and prediction of functional modules. The number of interactions associated with each of the nine datasets are provided. B: Datasets and network accuracy. The cumulative log likelihood score (LLS) was obtained from comparison with EcoCyc functional categories and provides a measure of accuracy associated with functional linkages. The relative contribution of each of the nine datasets to the LLS for each linkage is indicated. For the derivation of the definitive ‘functional’ network, we applied a threshold based on the LLS obtained for the small scale assays. Note this threshold exceeds the maximum contribution from any other single dataset. C: To assess the performance of the functional interactions we measured the precision, recall and area under the receiver operating characteristic curve (AUROC) across each COG functional category, for both the functional network and for a set of 100 randomly generated networks that possessed the same topology as the functional network [81] (see Text S1 - *Generation of random networks*). Colours are consistent with the colours provided by the COG website (http://www.ncbi.nlm.nih.gov/COG/grace/uni.html). D: Network statistics for five *E. coli* networks, three comparable networks for *S. cerevisiae* and a randomly generated network with equivalent properties to the combined network (number of nodes and interactions but not topology). ‘*E. coli* combined’, ‘functional’ and ‘Hu *et al.*, TAP’ networks are described in the main text. ‘*E. coil* extended’ is the initial set of 58,844 interactions obtained prior to applying a threshold cut-off. ‘*S. cerevisiae* functional’ and ‘experimental’ datasets were derived from [32] and [23] respectively. The ‘*E. coli* Hu et al. TAP (adjusted)’ and ‘*S. cerevisiae* experimental (adjusted)’ datasets were generated by randomly removing connections until their average node degrees were similar to the equivalent functional networks. E: Overlap of the functional network with three experimentally derived networks and a set of random networks. ‘*Hu et al. TAP*’ refers to the complete *Hu et al. TAP* dataset. ‘Filtered’ refers to the *Hu et al. TAP* network in which we removed interactions that also featured in the large scale TAP dataset and were included in the functional network. ‘Pull Down’ refers to the large scale pull down dataset [21], removing direct interactions that were included in the functional dataset. ‘Random: same topology’ refers to the average values of 100 random networks created with the same number of nodes and interactions as the “Filtered dataset” [81] (see Text S1 - *Generation of random networks*). ‘Direct’ indicates that the interaction is preserved between the two networks. Numbers indicate the distance of proteins in the functional network compared with those that directly interact in each of the other three networks. Error bars are negligible and are not shown for clarity. F: Interactions between COG functional categories. Numbers indicate the total number of interactions between each pair of COG functional categories. Colours represent Z-score deviations from the expected number of interactions. For further information see Text S1 - *Network analyses in the context of COG functional categories*.

Adopting a Bayesian framework, we constructed a high quality network of protein interactions for *E. coli* through the integration of interaction data from seven sets of computational predictions and three sets of experimentally verified interactions that include both large scale pull down and small scale assays (Fig. 1A and Table S1). Each dataset was assigned a log likelihood score (LLS) calculated from its performance relative to a gold standard set of functional annotations (see methods). Here we used EcoCyc [5] functional categories. Datasets with higher frequencies of interacting proteins that share a common functional category are assigned higher LLS's (indicating a higher confidence dataset). Other gold standard sets of functional annotations based on Clusters of Orthologous Genes (COGs) [29]; the Kyoto Encyclopedia of Genes and Genomes (KEGG) [30]; and Gene Ontology (GO) [31] terms were found to give comparable results (data not shown). Based on an analysis of dataset overlap (see Text S1 - *Generation of the functional network* and Fig. S1), we merged two highly redundant datasets and adopted a weighted sum scheme [32] to avoid potential biases due to data dependencies (Fig. S1E).

Integration of the datasets resulted in the scoring of 58,844 non-redundant functional linkages involving 4,149 (∼97%) *E. coli* proteins (Fig. 1B). Small-scale assays represent the most accurate datasets and were used to define a score cut-off for including interactions within our final dataset. The final high confidence network (hereafter referred to as the ‘*functional*’ network) contains 3,989 non-redundant linkages for 1,941 *E. coli* genes (∼45% of the *E. coli* proteome – Table S2). To assess the performance of the network reconstruction, we adopted a five-fold cross-validation scheme to predict membership of COG functional categories, using a label propagation method [33]. Comparisons of these predictions with previously assigned COG functional annotations revealed relatively high values of precision - TP/(TP+FP) - and recall – TP/(TP+FN) - (Fig. 1C). Of 19 functional categories, 15 had a precision in excess of 0.5 and 11 had a recall in excess of 0.5. Interactions involving proteins involved in cell motility (COG category N) demonstrated the best performance in terms of precision and recall (0.97 and 0.96 respectively). While interactions involving proteins involved in transcription (COG categories K) had among the lowest values (0.36 and 0.28 respectively) reflecting the tendency of these proteins to interact with and mediate a diverse range of cellular functions. Consistent with similar studies [27],[28],[32], we make the assumption that links between proteins from the same functional group are correct, while those that occur between different functional groups are incorrect. This assumption is supported by the large frequency of interactions derived from small scale assays, involving proteins annotated with the same COG or EcoCyc functional categories (Fig. S1F).

Applying the same cross-validation approach, we found that our functional network significantly out performs three previously published networks of *E. coli* functional interactions [15],[27],[28] (Fig. S2). Compared to these other datasets, our functional network demonstrated improved recall across all COG categories. Furthermore, the functional network provides the highest values of precision for eight of 19 COG categories and provides the next best value of precision for an additional eight categories. Finally, based on area under the receiver operating curve AUROC values, our functional network out performs the other datasets in 10 of 19 COG categories. For more discussion of how this network improves over previous analyses see Text S1 - *Comparisons with other datasets*.

### A combined network of functional and physical interactions yields novel insights into functional relationships

Recently a large scale PPI network consisting of 3,888 interactions derived for 918 proteins was generated for *E. coli* based on TAP [27] (Table S2). Since genome context methods were used to validate these interactions, it was not appropriate to include them as an additional dataset in our integration exercise. Instead, due to the reported high quality of these data we simply merged the *‘Hu et al. TAP’* dataset with our functional network to create a single *‘combined’* network of 7,613 interactions between 2,283 proteins. Graph analyses of all three networks (functional, *Hu et al. TAP* and combined) reveal the typical scale free properties associated with biological networks (Fig. S3). Comparisons of global topological metrics show how the significance cut-off impacts network node degree and shortest path lengths in the *E. coli* functional network. However, even accounting for differences in node degree, functional networks display higher eccentricities and betweenness values than their experimental counterparts, derived through the use of TAP (Fig. 1D). This is likely related to the tendency of TAP to identify interactions between common members of complexes that may not directly interact.

While the *Hu et al. TAP* and functional datasets share 557 proteins, only 241 (6%) of their interactions were common. When we consider indirect interactions within the functional network, we find that the overlap increases to 502 (13%) and 966 (25%) for path distances of two and three respectively – significantly greater than for randomly generated networks (Fig. 1E). This increase in overlap between the datasets arises as a consequence of the TAP approach identifying proteins through indirect interactions. Indeed, when we take into account these indirect interactions, we note that the overlap between the functional and *Hu et al. TAP* datasets is relatively high compared to previous analyses comparing the overlap between different interaction datasets [34],[35].

### Functional relationships within the combined network

The availability of a large network of well annotated genes facilitates the study of the topological properties both within and between different COG functional categories (Fig. S4). For example, proteins from COG category J (translation, ribosomal structure and biogenesis) and L (replication, recombination and repair) tend to be highly connected (high node degree), perhaps reflecting their tendency to occur in complexes, and central to the global network (high betweenness values) indicating their fundamental role to *E. coli*. On the other hand, while proteins from COG category N (cell motility) tend to be highly connected, they have low betweenness values but strikingly, high node and mutual clustering coefficients. This suggests that these proteins form highly integrated systems that operate in relative isolation to the rest of the network (e.g. flagella see below). Analysis of topological relationships between different COG categories (Figs. 1F & S5) are similarly revealing of functional relationships. For example, proteins from in COG categories D, M, O and U (Cell cycle control, cell division, chromosome partitioning; Cell wall/membrane/envelope/biogenesis; posttranslational modification, protein turnover and chaperones; and intracellular trafficking, secretion and vesicular transport respectively), all share high numbers of connections. This may reflect the need to tightly coordinate these processes for purposes of cell growth and division. Conversely, proteins from COG categories E, G and P (amino acid, carbohydrate and ion transport and metabolism respectively) are not highly connected and are also more distant (high shortest path lengths) to other COG categories, suggesting that these processes operate as functionally distinct modules within the global network.

### Organization of the combined network into functional modules

**Figure 2:**
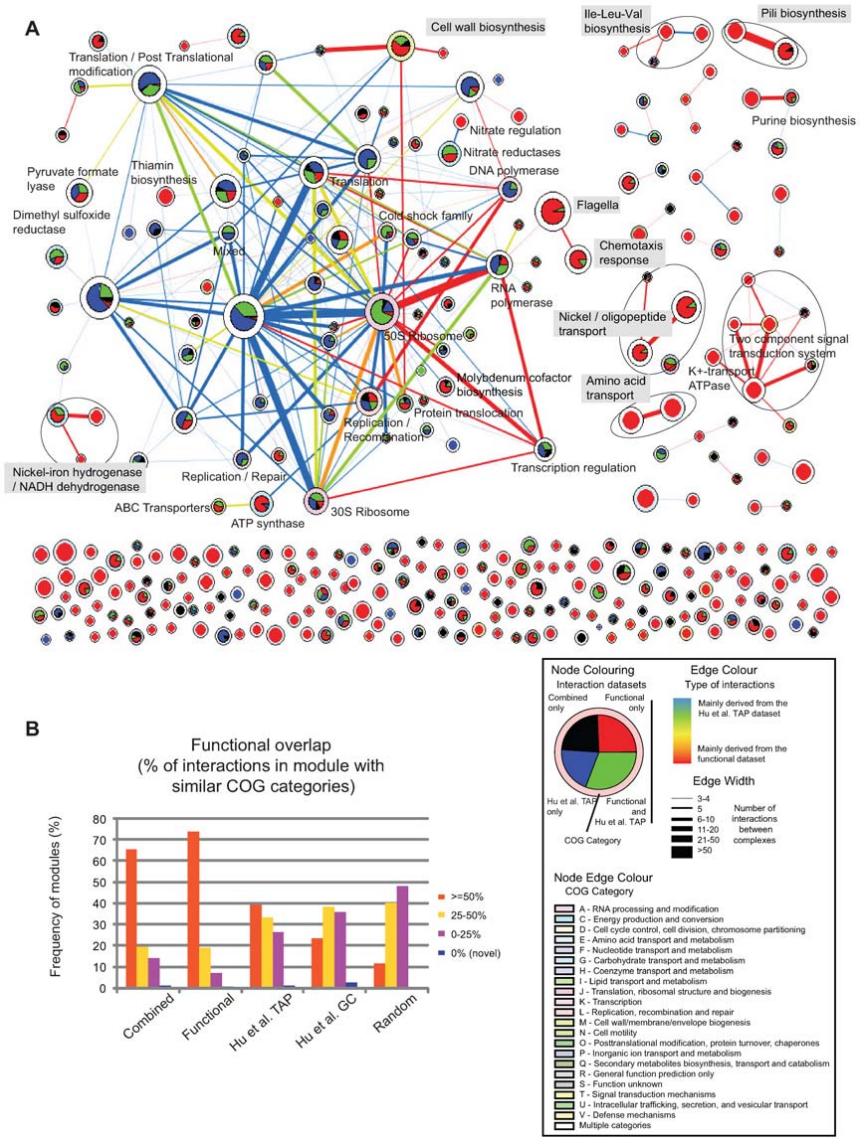
Organization of the combined *E. coli* protein-protein interaction network into functional modules. A: Graphical overview of 316 interconnected functional modules. Each pie chart represents an individual functional module, its relative size indicating the number of proteins in the module (only modules with 3 or more proteins are shown). The colours of each slice indicate the proportion of proteins found in functional modules predicted by either the functional, *Hu et al. TAP* or combined networks. Module borders are coloured if >60% of their members are associated with a single COG category (white otherwise). Edges represent *Hu et al. TAP* and/or functional interactions linking pairs of modules. Edge colours indicate the relative contribution of each network in the interaction. Edge thickness indicates the number of interactions between each module pair. B: Functional overlap of modules generated for the three networks presented in this study together with a previously published set of modules generated from a functional network (Hu et al. GC [27]) and 100 sets of modules generated by randomly swapping component genes between the modules generated from the combined network. Module overlap was determined through common membership of COG functional categories of their constituent proteins. Novel modules are defined as those in which component proteins are either not assigned a COG category or assigned the generic COG categories, S (‘Function unknown’) or R (‘General function prediction only’).

An emerging paradigm from the analysis of protein interaction networks is the tendency for protein activity to be coordinated through distinct functional modules. Applying the Markov cluster algorithm (MCL) [36] to the combined network, we identified 316 modules composed of three or more proteins (together with 243 two component clusters and 33 singletons – Fig. 2A and Table S3). 209 (66%) of the predicted modules (containing three or more proteins) possessed a high proportion (> = 50%) of common COG functional annotations (Fig. 2B and Table S3) and hence likely correspond with known functional modules such as protein complexes and biochemical pathways (see next section). Conversely we identified three modules that could be defined as novel. Finally 16 modules were composed of proteins with non-overlapping COG categories (ignoring the uninformative COG categories R, S or -). The heterogeneous nature of these modules, suggest that they may represent novel linking modules interconnecting different functional processes. Compared to the functional network, modules derived from the *Hu et al. TAP* network were more functionally heterogeneous, with only 39% of the predicted modules (containing three or more proteins) possessed a high proportion (> = 50%) of common COG functional annotations (Figs. 2B, S6 & S7 and Table S3). These differences are further exemplified by the higher proportion of inter-module∶intra-module interactions observed in the *Hu et al. TAP* network (2,329∶845 for the *Hu et al. TAP* network; 1,247∶2,107 for the functional network). This may reflect the tendency for TAP derived PPI data to include indirect interactions. From Fig. 2A we note the presence of a highly interconnected core of modules comprised predominantly of proteins derived from the *Hu et al. TAP* dataset. For the most part these are also linked through experimentally derived interactions. Modules derived mainly from the functional network are either isolated or tend to group into smaller discrete clusters of functionally related modules. Noteworthy, the networks presented here were functionally more homogeneous than a set of modules previously predicted from a network of functional interactions generated by Hu and colleagues [27] (Fig. 2B). The heterogeneous nature of this latter dataset reflects the high proportion of inter-module∶intra-module interactions (36,640∶19,043) that likely impact the resolution of the modules (see Text S1 - *Comparisons with other datasets*).

**Figure 3:**
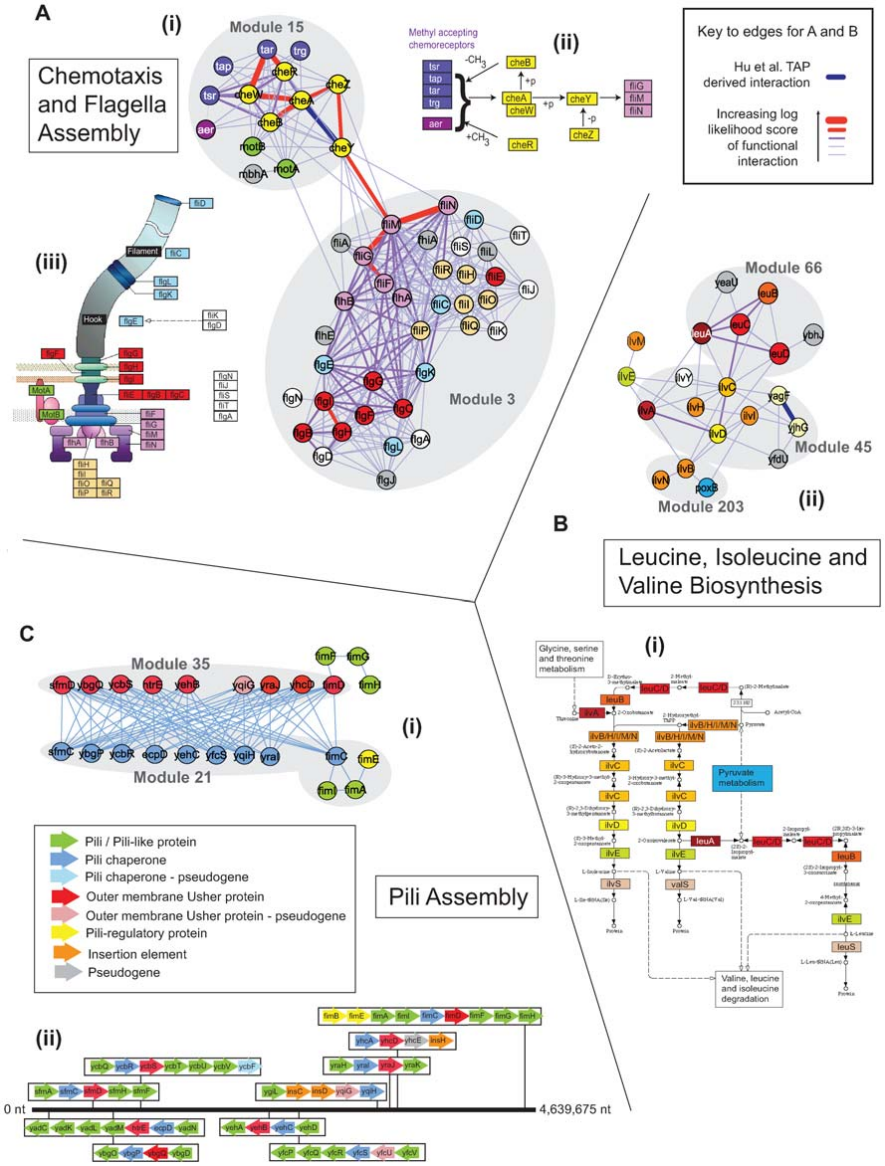
Examples of functional modules I. A: Chemotaxis and flagella assembly. (i) Within the combined network, components of chemotaxis and flagella assembly are organized within two distinct modules (3 and 15). Nodes are coloured according to their organization as defined by KEGG (see below); width of edges linking nodes indicate confidence associated with interactions. (ii) Map representing KEGG defined relationships associated with the chemotaxis pathway. (iii) Schematic of the structural organization of components of the flagella as defined by KEGG. B: Leucine, isoleucine and valine biosynthesis. (i) KEGG-based representation of Leucine, isoleucine and valine biosynthesis. (ii) Organization of components of the pathway within the combined network. Colours of nodes reflect KEGG pathway organization; width of edges linking nodes indicate confidence associated with interactions. C: Pili Assembly. (i) Two components of pili assembly are the outer membrane usher proteins and the pili chaperones. Within the combined network, family members of these proteins are organized into two modules on the basis of common patterns of interactions (21 and 35). Note that no member of either module interacts with a component from the same module. (ii) Linear representation of the operon organization of pili assembly proteins within the *E. coli*. Colours of nodes and genes in operons reflect functional roles (see inset).

Within the *E. coli* proteome, 1293 (∼30%) proteins have either not been assigned a COG functional category or assigned an uninformative category (S - ‘function unknown’ or R - ‘general function prediction only’). The organization of proteins into functional modules provides a valuable resource for further studies aimed at elucidating the functions of these poorly characterized proteins. For example from Fig. 3C below, we might infer that the uncharacterized protein yehB (annotated as a *putative outer membrane protein*) is involved in pili assembly. Interestingly, initial studies inferring functional annotations on the basis of common annotations within defined modules was found to be more accurate than one based solely on direct neighbour interactions (Fig. S6C and Text S1 - *Prediction of functional annotations for unknown genes*).

### Modules represent protein complexes, biochemical pathways and batteries of functionally and evolutionary related processes

**Figure 4:**
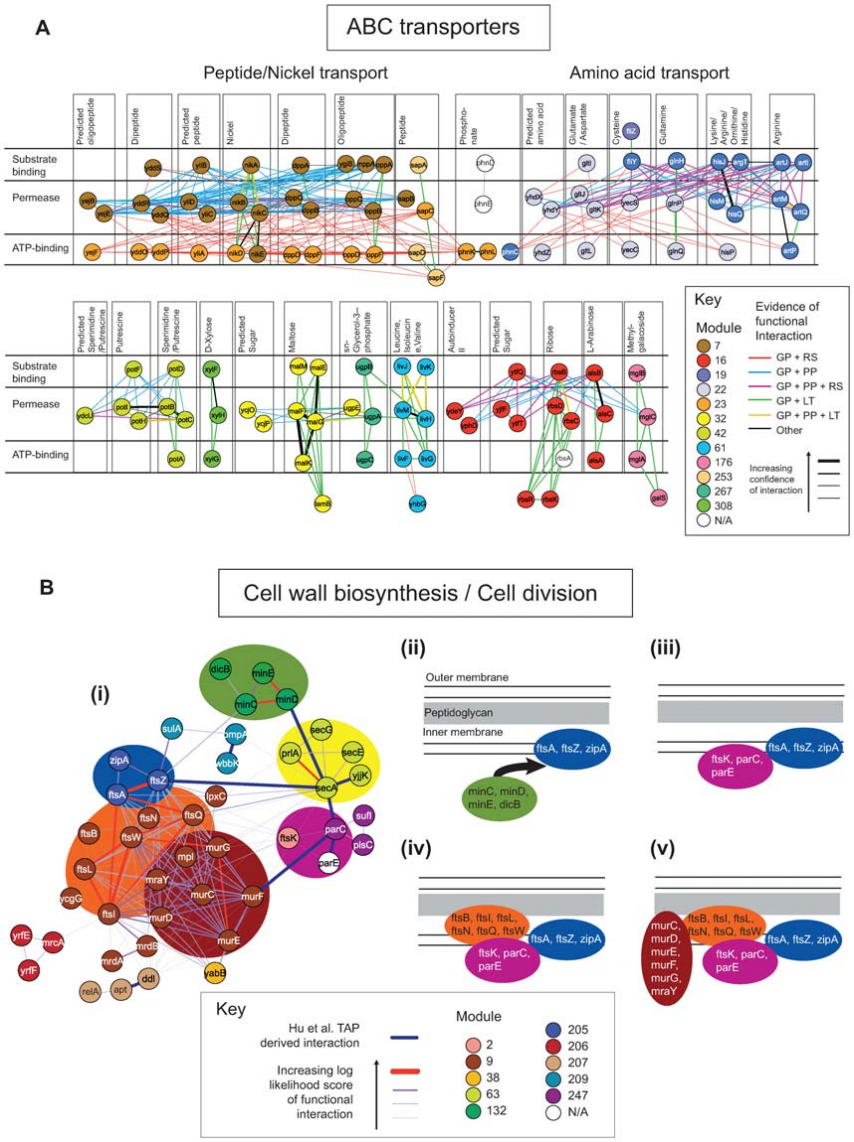
Examples of functional modules II. A: ABC transporters. Within the combined network, a number of modules were identified as containing components of ABC transporters, presented are a select 12, organized into substrate binding, permease and ATP-binding components as defined by KEGG. Colours of nodes indicate module membership (white nodes represent components that were not associated with one of the 316 modules). Colours of links represent type of supporting evidence (GP = genome proximity; RS = Rosetta stone; PP = phylogenetic profiles; LT = literature curation). B: Cell Wall Biosynthesis and Cell Division. (i) Subnetwork of 10 defined modules containing proteins associated with cell all biosynthesis and cell division. Nodes are coloured according to module membership. The larger background ovals indicate groups of proteins with common functional roles. (ii–v) Schematics illustrating the organization and operation of components during cell division. FtsZ is recruited to the site of cell division under the control of the minCDE, and subsequently recruits ftsA and zipA (ii). FtsK mediates the localization of components of TopoIV (parCE) required for chromosome partitioning, and is dependant on ftsA and zipA (iii). Further recruitment of additional cell division proteins – ftsBILNQW - (iv) is followed by the localization of cell wall biosynthetic machinery which includes members of the peptidoglycan biosynthesis pathway – murCDEFGY (v). Inclusion of secA interactions may be related to the fact that both secA and ftsZ both bind tightly to the inner membrane in the presence of MgCl2 [82].

While modules generated solely from physical interaction data are known to represent protein complexes [11],[19], those derived from functional interaction data may have other biological interpretations. Within the *E. coli* interactome, as well as known protein complexes (e.g. the 30S and 50S ribosomal subunits, RNA and DNA polymerases), we identified modules that represented biochemical pathways (e.g. nitrate regulation and cell wall biosynthesis) as well as batteries of functionally and evolutionary related processes. To illustrate the types of relationships that are associated with modules generated from mainly functional interaction data, we present several case examples of modules representing both well characterized and novel biochemical systems (Figs. 3 & 4). In these detailed views, interactions with different levels of confidence are presented. In general we find that proteins with interactions of lowest confidence scores are indicative of a general functional association (i.e. the protein forms part of the complex/pathway but its precise role is ambiguous). On the other hand, interactions with higher confidence scores may reflect closer functional relationships that can serve as a focus for more detailed investigation.

#### Chemotaxis and flagella assembly

Fig. 3A shows the interactions between modules 3 and 15 (Table S3) consisting of proteins involved in flagella biosynthesis and chemotaxis respectively. Within the flagella, most of the interactions with the strongest support (thick purple and red lines) are between components of the motor and the rod, ring and hook structures. Further investigation reveals that unlike the interactions with lower confidence scores, these interactions are additionally supported by phylogenetic profiling evidence, suggesting that these structures are co-inherited as distinct evolutionary units, consistent with a previous study that defined a core set of flagella genes [37]. The interactions with the strongest evidence involved fliFGMN and reflect experimental studies targeting the structure of the switch complex [38],[39]. Within the chemotaxis module, those interactions with the strongest support follow established pathways. For example the methyl-accepting chemoreceptor proteins (MCPs), tsr and tar, have strongest interactions with cheW, consistent with its role coupling MCPs to cheA [40]. Furthermore, interactions between chemotaxis and the flagella are organized through cheY which is known to control the direction of the flagella motor [41].

#### Leucine, isoleucine and valine biosynthesis

An example of a metabolic pathway is provided by modules 45, 66 and 203 which together comprise proteins involved in leucine, isoleucine and valine biosynthesis (Fig. 3B). Comparisons between the interactions involved in these modules with the KEGG defined pathway reveal that while leuABCD (organized as part of module 66) form a functional module associated with a branch of the pathway, other KEGG pathway relationships correlate less well with the interaction data (Fig. 3B). For example, there is strong evidence for functional links between ilvA and ilvD, and ilvC and leuC which do not appear close in the pathway. Compared to interactions with weaker evidence, these interactions are additionally supported by their conserved co-expression. This might suggest that these proteins play major roles in regulating substrate flux within the pathway. Interactions involving ilvBHIN reflect the formation of two different acetolactate synthases which demonstrate differential expression and activities: ilvB and ilvN form acetolactate synthase I, while ilvH and ilvI form acetolactate synthase III.

#### Pili biosynthesis

Modules 21 and 35 (Fig. 3C) comprise proteins from families of pili assembly proteins i.e. chaperones and outer membrane usher proteins respectively. The modules are defined on the basis of common inter-module interactions with components of the other module and interestingly, lack intra-module interactions, i.e. each component of module 21 has a similar pattern of interactions with components of module 35 and vice versa. Subsequently, the MCL algorithm has placed components with the similar patterns of interaction into the same cluster. These linkages are based on genome proximity and phylogenetic profiling methods. Their subsequent clustering into two distinct modules, correlates with their membership in two discrete gene families [42]. Pili assembly proteins form part of a set of related operons that include structural subunits that form mature pili [43]. While we might expect these structural subunits to form similar modules, their greater sequence diversity (as exemplified by their division into separate gene families) precludes the detection of their interactions via genome proximity and phylogenetic profiling methods. The grouping of assembly proteins represents a novel modular class reflecting a battery of functionally interchangeable elements, which in the case of these proteins allow the bacterial cell to attach to a variety of different surfaces important for colonization of host tissues in pathogenic strains [44].

#### ABC transporters

ABC transport systems are typically composed of three types of subunits: an extracellular substrate binding subunit; an intracellular ATP-binding subunit and a membrane incorporated permease [45]. The division of these related proteins into several modules (e.g. modules 7, 16, 19, 22, 23, 32, 42, 61, 176, 253, 267 and 308 - Fig. 4A) reflects both functional and evolutionary relationships. For example, components involved in seven nickel and peptide transport systems are associated with three modules. Interactions between the ATP-binding components and the permeases of the peptide/nickel and amino acid transporters are mostly supported through genome proximity and Rosetta stone methods. This is consistent with reports that gene fusion between permease and ATP-binding domains is a common feature of the ABC family [46] (albeit apparently restricted to these classes of transporters). As for the pili assembly proteins, the large number of shared interactions between the permeases and the ATP- and substrate-binding components is related to a high degree of sequence homology [46]. The relative isolation of other subsystems within this network (e.g. the D-xylose transporter), is a consequence of their relatively specialized functional roles and more distant evolutionary relationships.

#### Cell wall biosynthesis/cell division

In addition to inferring evolutionary relationships, inter-module interactions can also illuminate potential mechanisms of core cellular processes such as cell wall biogenesis and cell division (Fig. 4B) [47]. For example, cell division begins with the assembly of ftsZ into a ring structure followed by the recruitment of ftsA and zipA. This process is controlled by the Min system (via minC), dicB (also via minC) and sulA. Next, ftsK is co-localized which mediates the further assembly of additional cell division proteins: ftsBILNQW. In addition, ftsK plays an important role in chromosome partitioning including the decatenation of newly replicated chromosomes by TopoIV (which is composed of parC and parE) [48]. Cell wall biogenesis involving peptidoglycan biosynthesis is thought to occur at the site of cell division [49],[50]. The modular organization of the proteins in the peptidoglycan biosynthesis pathway with the cell division proteins, suggest that their recruitment may be mediated by ftsI and/or ftsW which possess strongest evidence of interaction.

#### Novel modules

In addition to well characterized systems, we also identified modules comprised of proteins which have not been assigned to an EcoCyc functional category (Table S4). A notable example is module 102, composed of five proteins: sspA; sspB; yraP; yrbD and ybaT. sspA is a global regulator that has been associated with acid resistance [51], while sspB is a ribosome associated protein that enhances the degradation of incomplete peptides when protein synthesis is stalled [52]. Little is known about yraP except that it is predicted to reside in the periplasm. The latter two proteins are putative transporters: ybaT is a member of the APC superfamily and its expression has been found to increase in response to acid stress [53]; while yrbD is a predicted ABC-type organic solvent transporter. Together these annotations suggest that these proteins form part of a stress response module. Further elucidation of their functions may emerge through focusing on their roles under exposure to acid and/or organic solvents.

### Evolution of the combined network and the integration of genes acquired through lateral gene transfer

The availability of a large scale interaction map for *E. coli* provides a valuable resource for exploring the evolution of protein interaction networks in bacteria. Consistent with previous studies in yeast and a smaller network derived for *E. coli* [9],[11], essential and/or highly conserved proteins are more highly connected and occupy more central roles within the combined network compared to non-essential and poorly conserved proteins (Fig. S8). Proteins from large gene families (>10 members) were also more highly connected and centric to the network (Fig. S9A). However, it should be noted that the number of connections associated with members of large gene families may be inflated due to the fact that they often possess similar phylogenetic profiles (one of the features used for generating the functional network). Nonetheless we also note that for each network, from 25–35% of genes from the same gene family have a shortest path length of two, indicating a common interactor. Together these findings highlight the role of conserved and essential proteins in coordinating cellular processes and support a model of preferential attachment in which duplicated proteins tend to interact with the partner of their paralog [54]–[57].

A unique facet of bacterial evolution is their ability to readily acquire new genes through lateral gene transfer events (LGT). Of 359 previously identified LGT genes [58], only 130 (36%) were identified within the combined network. On the whole proteins derived from these genes were poorly connected and found at the periphery of the network (Fig. S9B). For example, of the 130 LGT derived proteins in the network, 63 (48%) had a betweenness value of 0, i.e. they are connected to only one other protein, compared to 673 out of 2,173 (31%) for non-LGT proteins (χ^2^ = 17.26, p<0.0001, Chi-Square test). Similar results were obtained for both the *Hu et al. TAP* and the functional networks. These results suggests that LGT derived proteins largely contribute to PPI network evolution through the addition of peripheral functions, perhaps in response to changed environments [59]. For example, the peripheral (betweenness = 0) protein gadX is a regulator of two isoforms of glutamate decarboxylase which operate in several amino acid metabolic pathways. The predicted origin of gadX in *E. coli* through LGT suggests a recently acquired role in the network linking pH sensing with differential expression of these decarboxylases which are known to play a major role in acid resistance [60]. It is worth noting that the finding that LGT genes tend to occupy the periphery of networks, highlights a novel property that could be exploited for improving LGT detection methods.

**Figure 5:**
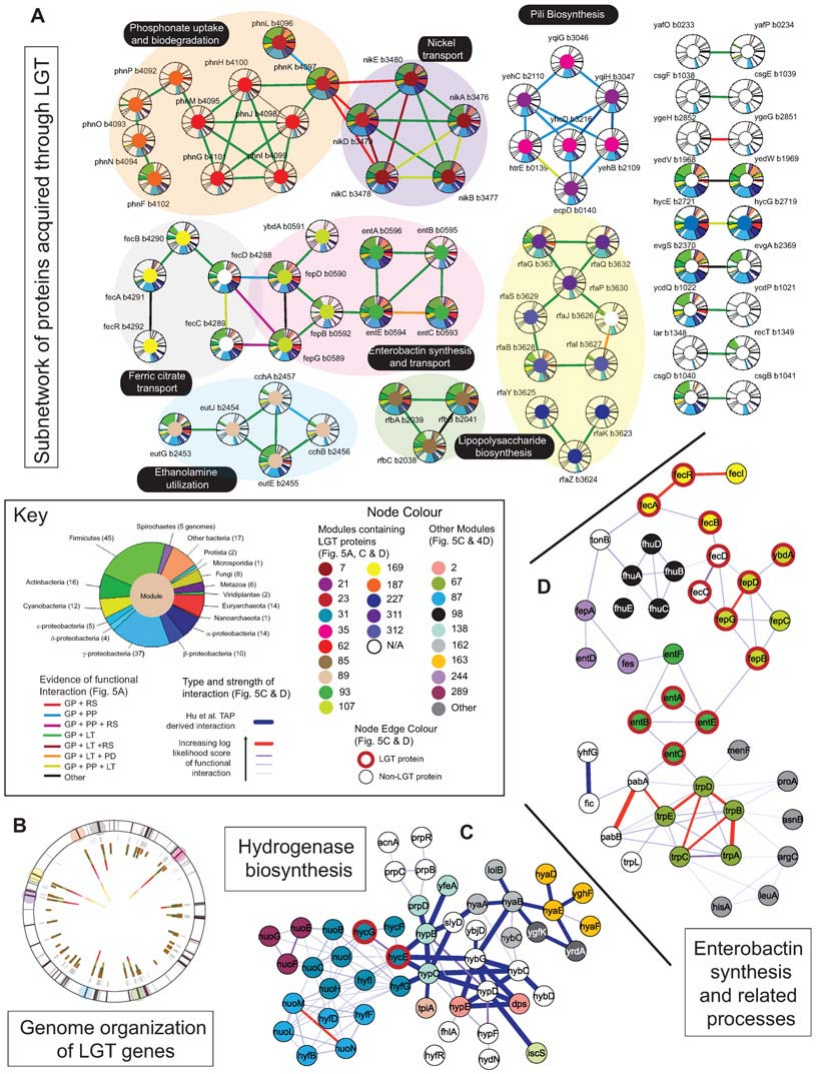
Organization of laterally transferred interactions. A: Organization of LGT-derived proteins within the combined network. Each pie chart indicates a single protein, with the coloured arcs reflecting its phylogenetic profile (see inset key). The colour at the centre of each pie chart indicates module membership. Large coloured ovals grouping proteins define gene neighbourhoods (each gene is within 2000 bp of at least one other gene). Colours of links represent type of supporting evidence (GP = genome proximity; RS = Rosetta stone; PP = phylogenetic profiles; LT = literature curation; PD = pull down). The embedded colour key indicates the breakdown of taxonomic groups used to construct the phylogenetic profiles – numbers indicate the number of genomes associated with each group. B: Organization of LGT genes with the *E. coli* genome. The outer circle indicates the location of LGT genes. Grey lines indicate LGT genes not identified within our network. Coloured lines extending into the center indicate LGT genes identified within our network, organized into gene neighbourhoods. Coloured circles indicate the relationship between the gene neighbourhoods and their organization within the network shown in A. C: Network organization of proteins involved in hydrogenase biosynthesis. Two proteins associated with hydrogenase 3, hycE and hycG are thought to derive through LGT and are highlighted. Also present in the combined network are proteins associated with: hydrogenase 1 (hyaABDEF); hydrogenase 2 (hybCDFO); hydrogenase 4 (hyfBDFGI); hydrogenase maturation (hypBCDEF and slyD); and NADH∶ubiquinone biosynthesis (nuoBCEFGHILMN). D: Network organization of proteins involved in enterobactin synthesis and related processes. Again proteins thought to derive through LGT and are highlighted. Also shown are components of the tryptophan biosynthetic pathway responsible for production of the chorismate precursor of enterobactin (trpABCDE); and components of two other related iron transport systems – fhuABCDE and fecABCD, which uptake iron via hydroxamate and dicitrate respectively.

Comparing shortest path lengths, we found a subset of LGT proteins that associate with other LGT proteins, although this appears to be a property of the functionally derived interactions rather than the *Hu et al. TAP* derived interactions (Fig. S9B). We identified a series of seven LGT specific subnetworks consisting of three or more interconnected LGT proteins with an additional nine other pairs of interacting LGT proteins (Fig. 5). In many cases, LGT genes associated with the same subnetwork were found in close genomic proximity, possessed similar phylogenetic profiles and were also organized within the same functional module (Figs. 4B and 5A), suggesting a mode of lateral evolution in which functional units may be co-inherited through discrete transfer events. For example, fourteen genes involved in phosphonate uptake and transport are organized into a single operon [61] and grouped into four functional modules, including module 62 which contains phnGHIJM. To examine the role of LGT in modular organization, we present two detailed examples involving hydrogenase and iron transport systems respectively.

The *E. coli* genome encodes four hydrogenases (Hyd1-4): Hyd1 and Hyd2 are isoenzymes involved in hydrogen uptake, while Hyd3 and Hyd4 perform the reverse reaction, although the physiological role of Hyd4 is not clear [62]. Hyd3 is encoded by hycBCDEFGHI, of which hycBCDEG are predicted to derive through LGT. Only three proteins associated with Hyd3 - hycEFG - are present within the combined network (Fig. 5C). Together these are organized in a single operon and form part of module 31 along with components of Hyd4 and NADH∶ubiquinone oxidoreductase, reflecting common sequence similarity relationships between the three systems [63]. Linking Hyd3 subunits to components of Hyd1 and 2, are a variety of proteins required for the maturation of the active hydrogenase enzymes, including hypBCDEF and slyD [62]. The emerging picture suggests that the putative acquisition of many components of Hyd3 via LGT and their integration into the network as a functional entity was facilitated by the presence of existing maturation proteins which were originally associated with Hyd1 and 2.

Enterobactin is a siderophore, produced by *E. coli* which is secreted by *E coli* and used to sequester and import iron and has been implicated in host invasion [64]. Evolution of metabolic pathways often involves the use of pre-existing metabolic precursors (note for example the links between enzymes from other amino acid pathways with those involved in tryptophan biosynthesis Fig. 5D). The synthesis of enterobactin requires chorismate produced by the enzyme trpD as a precursor and involves five enzymes: entABCEF. Of these, the first four are putative LGT genes organized in a single operon along with three of the four subunits (fepB, fepD, fepG) of the ABC transporter used to import ferric-enterobactin in a tonB-dependent process. Given a source of chorismate, the acquisition of these genes as a discrete functional module, provides the host bacterium with the ability to synthesise, secrete and import enterobactin. As an interesting aside, related genes in the pathway: entD, entF, fepA, fepC and fes, are also located in the same genomic proximity but were not predicted to have derived from LGTs. Finally it is worth noting the presence of two additional ABC-based iron transport systems within this subnetwork: fhuABCDE and fecABCD, responsible for the uptake of iron via hydroxamate and dicitrate respectively. While the fhu-based transporter appears native to *E. coli*, the fec-based system is another LGT acquired system. The interactions between the permease subunits fecCD and fepGD reflects their close evolutionary relationships and highlights the need for most bacteria to evolve and maintain a diverse battery of iron uptake systems as they attempt to compete with other microbial organisms for this relatively limited resource [65].

### Conclusions

**Figure 6:**
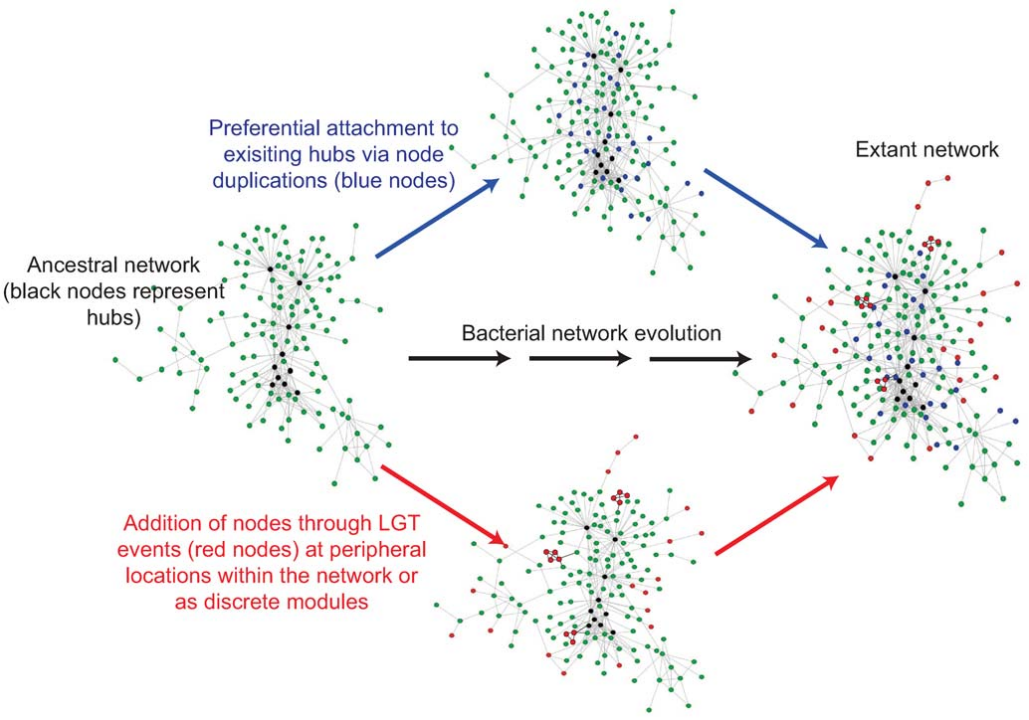
Amended model of the evolution of the *E. coli* interaction network. From an ancestral network, new interactions are acquired either through the duplication of existing genes (blue nodes) or the acquisition of novel genes through lateral gene transfer events (LGT – red nodes). The preferential attachment model suggests that duplicated genes are more likely to be located at the core of the network (genes associated with large gene families are more highly connected and more central to the network). On the other hand we find that LGT derived proteins tend to be more peripheral and/or integrated as a discrete module perhaps because they are less liable to disrupt essential functions associated with the network core.

Here we have combined a novel functional network with a recently generated experimental network to provide a global view of the modular organization of proteins in *E. coli*. The identification of functionally coherent modules, their interactions and the emergence of ‘neighbourhoods’ of interconnected modules represent a major step towards a deeper understanding of how biological processes are organized and operate. In an attempt to understand how the network may have arisen, we examined the role of gene family expansions and lateral gene transfer events on the generation of the network. From these analyses, we propose an amended model of network evolution (Fig. 6) based on preferential attachment as previously suggested [55]. In this new model, we suggest that the bacterial network gains interactions either through the duplication of existing genes, or through the acquisition of novel genes from LGT events. From the preferential attachment model and consistent with our analysis of gene family relationships we note that gene duplication events result in preferential growth at the core of the network. On the other hand, perhaps due to their potential to disrupt essential interactions that are enriched in the core of the network, the acquisition of new interactions through LGT events occurs mainly at the network periphery. Instead, the evolution of the network through LGT events at the network periphery might be associated with contingency genes allowing the bacteria to adapt to new ecological niches. It should be noted that the LGT derived proteins used in this study were detected mainly by their composition properties and may therefore be biased towards more recent transfers [58]. It cannot therefore be discounted that proteins derived through older LGT events that are less easily recognized, may have become integrated into the network, potentially developing into core components of the network.

Previous studies of PPI networks, have shown that many functional modules tend to be conserved over evolution [66],[67]. More recently, studies of protein complexes evolution suggest that protein complexes form early in evolution and evolve as coherent units [68] and that duplication of self-interacting proteins play a key role in their formation [69]. Here we expand on these ideas by suggesting that at least in bacteria, LGT events resulting in the simultaneous acquisition of several functionally related genes may also contribute to the formation of a modular network structure.

To our knowledge, this network represents the most comprehensive and accurate gene network reconstruction in *E. coli* that not only provide insights into the evolution and organization of bacterial protein interaction networks, but may be usefully exploited to help understand the molecular basis of pathogenesis. Furthermore, the identification of groups of proteins organized into discrete functional modules will assist the design and construction of artificial biological systems and hence provide a valuable contribution to the emerging field of synthetic biology [70],[71]. To allow researchers to freely download explore these datasets a publicly available web tool has been developed - http://www.compsysbio.org/bacteriome/ [72].

Finally, it is important to note that the network presented here represents only 45% of the *E. coli* proteome. While the coverage of the network will improve as additional datasets become available, we would nonetheless encourage researchers, interested in genes not contained within this dataset, to explore the other previously published datasets outlined in this paper. Links to these datasets are also provided on our project website.

## Methods

### Sources of data

Datasets to derive the functional network included seven computational (*C*) and three experimental (*E*) datasets: Phylogenetic profiles (*C*) [14]; Rosetta stone (*C*) [14]; Gene neighbourhood (*C*) [14]; Gene clusters (*C*) [14]; Literature curated (*C*) [22]; Interologs of *H. pylori* (*C*) [12],[73]; Conserved coexpression (*C*) [13]; Large scale TAP [9] (*E*) and Small scale assays (*E*) from DIP [74],[75]; and Large scale pull down (*E*) [21]. Numbers of proteins and interactions associated with each dataset are presented in Table S1, along with a breakdown of the experimental methods used to derive the small scale assay dataset. Each dataset assumed a bait∶prey (“spoke”) model of interaction (as opposed to a “matrix” model, in which each component of a complex is assumed interact with all other components of the complex). Due to the high level of overlap between the Gene neighbourhood and Gene clusters datasets, they were combined into a single set of interactions termed Gene proximity. In order to test the correct assignment of functional linkages we used four different benchmark sets: EcoCyC [5], the Clusters of Orthologous Group (COG) [29], the Kyoto-based KEGG [76], and the Gene Ontology (GO) annotation database [31]. Our analyses also incorporated a recently generated large scale TAP-derived network (designated *Hu et al. TAP*) containing 3,888 interactions between 918 proteins [77]. This dataset also assumes a spoke model of interaction. Due to the high quality and coverage of this data set which has also been subjected to validation through a similar data integration process, it was not included in the generation of the functional network. Instead the two networks (*Hu et al. TAP* and functional) were merged into a single combined network featuring 7,613 interactions between 2,283 proteins. Lists of essential and non-essential proteins were derived from Zhang and co-workers [78]. For further details on these datasets see Text S1 - *Methods*.

### A probabilistic method for integrating functional genomics data

To derive a high quality dataset of functional interactions, information from the seven computational and three experimentally derived datasets were integrated within a Bayesian framework. The scoring scheme used in this study derives from Bayesian statistics and is similar to that used by Lee and co-workers [32], in which each input data set, either experimentally or computationally derived, adds some evidence that a pair of genes are functionally linked. Each experimental and computational data set is evaluated for its ability to reconstruct known pathways by measuring log likelihood scores (LLS) representing the likelihood that a pair of genes are functionally linked.
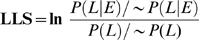



*P(L|E)*/∼*P(L|E)* represents the *posterior* odds ratio, where *P(L|E)* represents the frequency of interactions *(L)* in a dataset *(E)* between proteins participating in the same functional category (as defined by EcoCyc); ∼*P(L|E)* represents the frequency of *L* in *E* participating in different functional categories. *P(L)*/∼*P(L)* represents the *prior* odds ratio, where *P(L)* represents the frequency of interactions between all *E. coli* proteins participating in the same functional category; and ∼*P(L)* represents the frequency of interactions between all *E. coli* proteins participating in different functional categories. Higher values of LLS indicate more confident interactions associated with the dataset.

To derive a score associated with a functional interaction, we integrate the LLS's from each dataset in which that interaction is found. To examine potential biases that may arise from data dependencies we applied a weighted sum scoring scheme [32] to derive a final score *S* associated with each interaction:
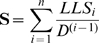
where *LLS*_i_ represents the LLS for the functional interaction from dataset *i* (ordered by descending magnitude of the *n* log likelihood scores for the given interaction); *D* is a free parameter representing the relative degree of dependency between various datasets; and n is the number of datasets containing the interaction. Here we examined values of *D* from 1 to ∞ and found that *D* = 1 gave the best performance in terms of accuracy (LLS) and coverage, suggesting that the datasets were independent (Figure S1E). Hence in this study, the final score for a functional interaction was simply derived from the sum of LLS's of all datasets in which the interaction was found.

Finally a cut-off based on the LLS derived from the small scale experimental dataset was used to define a high confidence set of functional interactions (Fig. 1B).

### Assessment of the performance of functional interactions

To assess functional interactions we used a previously published label propagation method using a threshold cut-off > = 0.5 [33] with five-fold cross-validation based on COG category assignments. We derived values for precision (true positives/(true positives+false positives)), recall (true positives/(true positives+false negatives)) and area under receiver operator characteristic curve (AUROC) from 100 replicate samplings.

### Network analyses

Network statistics were derived using in house perl scripts and the two software packages: Pajek (http://vlado.fmf.uni-lj.si/pub/networks/pajek/) and tYNA (http://tyna.gersteinlab.org/). The global network view was generated using an in-house Java based applet. Other network views were generated using Cytoscape (http://www.cytoscape.org/). The genome ideogram was generated using the Circos software (http://mkweb.bcgsc.ca/circos/?home).

Functional modules were predicted using the Markov clustering algorithm [36], testing several inflation parameters and using values that provided the best overlap of the computed clusters with COG functional categories (Fig. S6). Note the average % overlap in COG categories across modules derived from the combined network was found to only vary between ∼56–59%. Hence, even without optimization, there is a high proportion of clusters with common COG terms.

### Conservation analyses

For each *E. coli* sequence, a BLASTP [79] search was performed against each of 199 different organism genome data sets derived from the COGENT database [80] (Table S5). Homologs for each protein were determined based on a raw bit score threshold of 50. These homologs were used to generate the phylogenetic profiles presented in Fig. 5A. For additional conservation analyses, two sets of conservation were defined. The first consists of three categories: conserved (homologs in more than 100 genomes - 932 proteins in the combined network); medium conserved (homologs in at least 25 genomes – 539 proteins); and non conserved (homologs in less than 25 genomes – 553 proteins). The second set consists of eight categories: *E. coli* specific (no detectable homologs outside *E. coli*– 21 proteins in the combined network); gammaproteobacterial specific (no detectable homologs outside gammaproteobacteria – 121 proteins); proteobacterial specific (no detectable homologs outside proteobacteria – 129 proteins); and proteins with detectable homologs to 1–5 different groups of prokaryotes (Cyanobacteria, Spirochaetes, Firmicutes/Actinobacteria, ‘Other bacterial groups’ and Archaea – Table S5) as defined by the NCBI taxonomy resource (http://www.ncbi.nlm.nih.gov/Taxonomy/taxonomyhome.html/) – 110, 186, 249, 433 and 822 proteins associated with 1,2,3,4 and 5 groups respectively. We considered a functional interaction to be preserved in a genome if both interacting proteins have detectable homologues.

Expanded descriptions of network generation and analyses are provided in the supplementary files - Text S1, Figures S1, S2, S3, S4, S5, S6, S7, S8, and S9, and Tables S1, S2, S3, S4, S5, and S6.

## Supporting Information

Text S1 Supplementary Information
(0.07 MB PDF)

Figure S1 Dataset integration and functional overlap. (A and B) Overlap between the data sets used for inferring functional interactions. In an initial approach ten methods were integrated via a Bayesian framework to predict functional interactions. (A) shows the percentage of these interactions supported by one method that are also supported by each of the nine other methods. In the definitive approach used to derive the ‘functional network’ described here, we combined the gene cluster and gene neighbourhood data sets into a single non-redundant set (genome proximity). The data sets were then reanalyzed to predict 3989 interactions between 1941 proteins. The graphic in (B) shows the percentage of these new interactions supported by one method that are also supported by each of the other eight methods. Background colour scales from the highest level of overlap (red) to the lowest (blue). TAP = Tandem Affinity Purification; CO = Conserved Co-expression; PD = Pull Down experiments; RS = Rosetta Stone; LT = Literature mining; SS = Small Scale experiments; PP = Phylogenetic profiles; GC = Gene Cluster; GN = Gene Neighbourhood; GP = Genome Proximity (see above); and IN = Interologs (see supplemental methods for sources of data). Number of functional linkages inferred by each method is given in brackets. (C) Number of interactions in the functional network supported by one method that are also supported by each of the eight other methods. Background colour scales from the highest values (red) to the lowest (blue). (D) Breakdown of the number of methods used to support each of the 3989 interactions in the functional network. Note the 158 interactions supported by a single method were derived from the small scale assays that did not have any extra supporting evidence. (E) To examine potential biases that may arise from data dependencies we applied a weighted sum scoring scheme [33] to derive a final score S associated with each interaction (see methods in main text). D is a free parameter representing the relative degree of dependency between various datasets. Here we found that D = 1 gave the best performance in terms of accuracy (LLS) and coverage for our functional network, suggesting that the datasets used in this study were independent. (F) Network accuracy for four networks: the functional network; a network derived from small scale assays and two randomly generated networks (the ‘shuffled’ network was created by randomly reassigning interactions within the functional network, the ‘random’ network was created by randomly selecting an equivalent number of proteins from the E. coli proteome and randomly assigning an equivalent number of interactions). The bars represent the percentage of interactions in each network in which both proteins share the same functional category assigned by either COG or EcoCyc [19],[20]. Error bars indicate standard deviation for 30 replicate random or shuffled networks (Text S1 - Methods).
(0.46 MB PDF)

Figure S2 Comparison of the functional network to three previously published functional networks. (A) Overlap of interactions derived from four E. coli functional interaction datasets: Functional (this study); String [4]; Hu et al., GC [5]; and Yellaboina [2]. Numbers in brackets indicate the total number of interactions associated with the dataset. (B) Sample precision-recall curves for four selected COG categories. Precision/Recall values were obtained using the five fold cross-validation method of assigning COG categories based on label propagation described in the main text. Different values of precision and recall were generated from increasing the threshold cutoff for label propagation from 0 to 1. (C–E) Measures of performance for the four functional datasets: (C) Area under the receiver operating characteristic curve (AUROC = sensitivity vs. (1-specificity)); (D) Recall; and (E) Precision. To maintain consistency across all COG categories, for graphs D and E (summarizing differences in Recall and Precision) we used a single threshold cutoff for label propagation of 0.5. Points and error bars represent the means and standard deviations obtained from 100 replicates. The functional network presented in this study significantly out performs the other three datasets in terms of improved recall across all COG categories. Furthermore, it provides the highest values of precision for 8 COG categories and the next best value of precision for an additional eight categories. Finally, in terms of AUROC values, our functional network out performs all three other datasets in 10 of 19 COG categories.
(1.58 MB PDF)

Figure S3 Scale free behavior of the networks. Node degree distributions of the four networks (combined, Hu et al. TAP, high- and low-confidence functional networks) presented in the paper. Each graph is a log-log plot of the number of interactions (k) for each protein as a function of frequency (p(k)). Each network demonstrates scale free behavior as shown by the linear relationships within each graph.
(0.38 MB PDF)

Figure S4 Distribution of topology measures for different COG functional categories within the combined network. (A) Distribution of betweenness values for each COG functional category. (B) Distribution of shortest path length between two proteins in the network calculated both for proteins from the same COG category and for proteins to other COG categories. (C) Distribution of node clustering coefficients for each COG functional category. (D) Distribution of mutual clustering coefficients for interactions involving both proteins from the same COG category and for proteins from different COG categories. (E) Distribution of node degrees for each COG category. Descriptions of COG category codes are provided in (E).
(1.76 MB PDF)

Figure S5 Topological relationships of COG functional categories within the three derived networks. (A) Number of interactions in the Hu et al. TAP and functional network between each pair of COG categories. Each combination of COG categories is coloured according to the significance (Z-score) of enrichment (red) or depletion (blue) of interactions compared with values obtained from 100 randomly generated networks. (B) Shortest path length between COG categories in the combined, Hu et al. TAP and functional networks. COG category combinations are coloured by the deviation of their shortest path length from the average for the network (red = enrichment, blue = depletion). COG category codes for (A and B) as shown in (C). ‘multi’ = proteins assigned to multiple COG categories. (C) Description of COG functional categories and numbers of proteins in each category associated with each network. Colours were obtained from the COG website (http://www.ncbi.nlm.nih.gov/COG/).
(2.33 MB PDF)

Figure S6 Performance of the MCL algorithm on module prediction. (A) Bars represent the average percentage of overlap in COG categories within module predictions at different MCL inflation values for the Hu et al. TAP, functional and combined networks. The percentage of overlap for each module was obtained by considering only the most abundant COG category in the module. (B) Size distribution of modules. Values represent the distribution of module sizes (log scale) for the Hu et al. TAP, functional and combined networks. The average for 100 random networks of similar size to the combined network are also shown. Lines of best fit and correlation coefficients (R-squared) are indicated for each data set. Note the relative steepness of the line associated with the random network compared with the other three networks. (C) Accuracy of functional network to predict correct COG annotations for two network-based methods: ‘neighbour linkage’ and ‘functional module’ using a leave-one-out cross-validation procedure (see Text S1 - Methods). Bars indicate the frequency of correct COG assignment. Two measures of stringency were employed: high stringency indicates that the majority of interaction partners/module members had the same COG category; low stringency indicates that any of the interaction partners/module members had the same COG category. In both cases correct COG assignment additionally required at least 20% of the interaction partners/module members to have the same COG category. Due to the difference in module size distributions (B), only the neighbour linkage method was applied to random and shuffled networks of equal size to the functional network. Error bars indicate standard deviation for 30 replicate random/shuffled controls.
(0.37 MB PDF)

Figure S7 Networks of predicted functional modules. Each graph indicates a network of predicted modules for the combined, Hu et al. TAP and functional networks. Each pie chart shows the proportion of proteins associated with each COG functional category (see inset for colour key). The size of the pie chart indicates the number of proteins associated with each module. Links between modules indicate interactions between proteins in different module. Note the greater functional heterogeneity associated with the Hu et al. TAP modules compared with the functionally derived modules.
(0.99 MB PDF)

Figure S8 Network properties associated with gene conservation and essentiality. (A and B) Graphs comparing network properties (node degree and betweenness centrality) with a protein's essentiality (A) and conservation (B). Conserved proteins are defined as those with homologs in more than 100 genomes (of 199), medium conserved proteins are defined as those with homologs in 25 to 100 genomes and non conserved proteins are defined as those with homologs in less than 25 genomes. Lists of genomes are provided in Table S5. Descriptions of how network metrics are calculated are provided in Text S1 - Methods. For each graph, results are provided for both the high quality (HQ) combined network (2,283 proteins, 7,613 interactions) and a lower quality (LQ) network consisting of the extended network of functional interactions together with the Hu et al. TAP network (4,190 proteins, 60,241 interactions). The inclusion of the low quality network in these analyses which reveal similar trends to the high quality network demonstrates that our results are not influenced by the large number of false negatives associated with the high confidence network. Also shown are results for a random network constructed with the same proteins and topology as the combined network [27] (see Suppl. methods). (C) Relationship between protein essentiality, hub-non hub interactions and shortest path lengths. Hubs are defined as proteins with more than 10 interactions; non-hubs are defined as proteins with less than three interactions. Again, results for a random network constructed with the same proteins and topology as the combined network are also shown. (D) Relationship between the node degree, betweenness centrality and node clustering coefficient of a protein and its degree of conservation within prokaryotes. Each protein in the combined network is assigned one of eight conservation categories: E. coli specific; gammaproteobacterial specific; proteobacterial specific; and 1–5 other prokaryotic groups (see Text S1 - Methods).
(0.75 MB PDF)

Figure S9 Network properties associated with gene family membership and laterally transferred genes. (A) Graphs comparing network properties (node degree, betweenness centrality and shortest path length) with gene family membership for the three networks. For the graphs relating betweenness centrality and shortest path lengths for the combined network, also included are the results from 100 ‘random’ networks sharing the same degree distribution as the combined network. The bottom graph indicates the frequency of proteins that interact (according to the combined network) with different numbers of proteins from the same family. Protein families were obtained with reference to the COGENT++ database [33]. (B) Graphs comparing network properties (betweenness centrality and shortest path length) with the origin of a protein (LGT versus non-LGT) for the three networks presented in this study. For each network, LGT genes tend to have lower values of betweenness indicating their peripheral position within the respective networks. The two tailed distribution of shortest path lengths observed for the functional network, highlights the finding that a proportion of LGT genes within this network, occur within discrete interconnected modules.
(0.70 MB PDF)

Table S1 Sources of data used to derive the functional network. This table lists the number of proteins and interactions associated with each dataset. Also presented is a breakdown of the number and type of experiments associated with the small scale assay dataset.
(0.02 MB XLS)

Table S2 List of interactions used to derive the combined network. The functional and Hu et al. TAP networks were combined into a single network. Locus ids, gene names, COG categories (“-” indicates no COG assignment) and description of gene products were obtained from the COG database [20]. ‘LLS’ refers to the likelihood scores obtained from the functional network. ‘Confidence scores’ refers to scores obtained for the Hu et al. TAP network [33]. The presence of LLS and Confidence scores for the same interaction indicates that the interaction was detected by both methods, otherwise the interaction was identified in only a single data set. COG category codes are provided in Fig. 1C.
(2.20 MB XLS)

Table S3 Functional module predictions for the combined, functional and Hu et al. TAP networks. Functional modules were predicted by using the MCL algorithm (see Methods). Locus ids, gene names, COGs categories (“-” if there is not COG assigment) and annotation description were obtained from the COGs database [20]. COG category codes are provided in Fig. 1C. EcoCyc annotations were obtained from the EcoCyc database resource [19].
(0.70 MB XLS)

Table S4 Enrichment of COG functional categories in modules derived from the three networks. Functional modules were predicted using the MCL algorithm (see Methods). For each module the major COG category was determined as the category assigned to the most proteins in that module, the % of proteins annotated with the major COG category is indicated. Module size indicates the number of proteins associated with the functional module. P-values were calculated based on expectation using 10000 random modules of equal size. * = p-value <0.1; ** = p-value <0.01; *** = p-value <0.001.
(0.09 MB XLS)

Table S5 List of genomes used for comparative analyses. List of full sequenced genomes analysed in the study obtained from the COGENT database [33]. Species are ordered by major taxonomic groups (Archaea; Bacteria; Eukaryota) and are also coloured by minor taxonomic groups. For each species its COGENT id and the number of sequences associated with the genome are given.
(0.06 MB XLS)

Table S6 Enrichment of essential proteins in modules derived from the combined network. Functional modules were predicted using the MCL algorithm (see Methods). Module annotation was provided if the overlap of COG categories among the module components was more than 60%, otherwise the module was assigned the COG code – ‘S’ (unknown). COG category codes are provided in Fig. 1C. Size represents the number of components within the module. E/NE(%) represent the percentage of essential/non essential components within the module. 42 essential functional modules were predicted at p-value <0.1. P-values were calculated based on expectation using 10000 random modules of equal size. * = p-value <0.1; ** = p-value <0.01; *** = p-value <0.001.
(0.06 MB XLS)
