# Supplementary material for: The Modular Organization of Protein Interactions in Escherichia coli
Source: PLoS Comput Biol. 2009 Oct 2;5(10):e1000523. doi: 10.1371/journal.pcbi.1000523 (PMC2739439; doi:10.1371/journal.pcbi.1000523)
Supplement: Text S1 — Supplementary Information (0.07 MB PDF) [file pcbi.1000523.s001.pdf]

SUPPLEMENTAL INFORMATION

Generation of the functional network

In an initial approach, we included two sources of gene proximity information – gene neighbourhood and gene cluster. However, since Bayesian integration assumes data independency, we examined the degree of overlapping of pairs of datasets in an all vs. all data set comparison. The highest overlap (~50%) was obtained by the gene cluster against the gene neighbour data set (Fig. S1A). Given that both methods use a measure of genome proximity to predict functional linkage, this high overlap indicates a high degree of dependency (redundancy) between both data sets. We therefore combined both data sets into a single non-redundant data set termed genome proximity, and recalculated the degree of overlap between the nine remaining data sets (Fig. S1B). Few comparisons produced non-overlapping functional linkages at all (for example the Tandem affinity purification (TAP) tagging versus the Rosetta stone data set, or the conserved-coexpression versus the interologs set). Interestingly, the maximum degree of overlap with the Small Scale data set was obtained by the Gene Proximity set (~25%), followed by TAP tagging (~14%), and Literature mining (~13%) data sets. Assuming the small scale assays provide the highest quality interactions, these results suggest that genome proximity and TAP-tagging as the best computational and experimental approaches, respectively, for the reliable prediction of functional interactions.

An analysis of the degree of contribution of all the functional genomics data sets analysed in this study to the overall performance of our probabilistic functional *E. coli* network shows that the combination between the Gene Proximity and the Literature-mining methods provided the highest number of functional interactions (2,040) (Fig. S1C). The literature-mining data set provided the highest number of functional interactions (240) supported by small scale experiments. The conserved-coexpression and the Interologs data sets were the only two not to share any functional interactions.

The final *functional* network contained a total of 3,989 interactions between 1,941 proteins (~45% of the *E. coli* proteome). Note the incorporation of predictions based on genome context methods reduces the potential for bias that have previously been associated with experimentally derived datasets [1]. The final network has an accuracy (LLS = 3.49) similar to the one obtained by small scale assays (LLS = 3.61) (Fig. 1B) but with a much higher coverage (46% and 11%, respectively) validating the utility of our approach. None of the functional linkages are contained in seven or more of the experimental and computational data sets. Only five linkages are represented by six data sets: cheY-cheA (LLS = 13.32); sucC-sucD (LLS = 12.88); pabA-pabB (LLS = 12.74); rpoA-rpoB (LLS = 11.43); and mobA-mobB (LLS = 11.43). 3,831 linkages (96% of the high confident network) are represented in at least two individual sets (Fig. S1C and D).

Comparisons with other datasets

Currently three other functional networks are available for *E. coli* – one generated by Yellaboina and co-workers (Yellaboina) [2]; one generated as part of the STRING database resource (STRING) [3,4]; and one generated by Hu and co-workers (Hu et al. GC) [5]. All three datasets

were generated by a similar Bayesian integration approach to that applied here. Nonetheless, the functional network provided here provides conceptual advances over these other datasets.

(1) Compared with the Yellaboina and Hu et al. GC datasets, we include many more datasets (Yellaboina used three genome context datasets, Hu et al. GC used four genome context datasets, here we used ten, including seven theoretical and three experimental datasets). The STRING database on the other hand does not focus on any single organism, but provides interaction data from 630 organisms. Due to licensing restrictions, it isn't clear which datasets are used for the generation of the *E. coli* interactions provided by the STRING database. However, website views suggest at least eight including 3 genome context methods, co-expression data (compared with the *conserved* co-expression dataset used here), experiments, databases, text mining (using an method developed in house) and homology searches. However it is not possible to get the details on which datasets are being used without going through the licensing agreements.

(2) All three datasets assume independence for the different data sources. This may be particularly problematic for the Hu et al. GC dataset which combined two alternative sources for determining the natural chromosomal association of bacterial genes in operons. In our analyses of dataset overlap, we noted significant overlap between the gene neighbourhood and gene cluster (operon) datasets, which would bias the scoring. We therefore merged these two genome context methods into a single *gene proximity* set. Similarly, such data independency cannot be assumed for gene neighbourhood and co-expression datasets due to the fact that genes in the same operon are co-transcribed (hence our use of *conserved* co-expression datasets).

(3) Here we use a gold standard set of interactions based on small scale experiments to benchmark our set of 58,844 interactions to derive a more limited set of ~4,000 high confidence interactions. The Yellaboina paper does not describe such a high confidence set and focuses on the analysis of the entire dataset of 78,122 interactions. The STRING interactions are scored based on comparisons to KEGG pathways. An arbitrary cutoff of 0.7 was used to define highly confident interactions. The Hu et al. GC interactions used the same scoring model as the STRING dataset, but rather chose a slightly higher but nonetheless arbitrary cutoff of 0.8 to define highly confident interactions.

(4) In comparing the overlap between the four datasets (Fig. S2A), we note that our functional network includes 406 interactions that are not present in any of the other three datasets. In addition, our functional network includes 758 interactions that are present in only one other dataset.

(5) Five fold cross-validation experiments, using COG categories as a benchmark (Fig. S2B-E) reveals that the functional network out performs the other three datasets in terms of recall across all COG categories. Furthermore, in terms of area under the receiver operating characteristic curve (AUC) values, our functional network out performs all three other datasets in 10 of 19 COG categories. Finally, the functional network provides the highest values of precision for 8 COG categories and provides the next best value of precision for an additional eight categories.

(6) Unlike the STRING dataset, there are no license restrictions on our datasets which we make freely available online for other researchers to download and use as they see fit (http://www.compsysbio.org/projects/bacteriome).

(7) Finally, module predictions performed on the Hu et al. GC dataset were found to be more functionally heterogeneous than the modules predicted for the functional network presented in this study (Fig. 2). This is likely associated with the higher proportion of inter-module:intra-module interactions associated with the Hu et al. GC dataset, that impact the functional resolution of the modules. For example, module 3 from Hu et al. GC consists of 71 proteins. Of these 33 are involved in flagella biosynthesis, all of which are associated with module 3 presented in the current study, which contains an additional 3 flagella biosynthetic proteins. In addition, Hu et al. GC module 3 also contains 13 proteins involved in chemotaxis response (represented as a separate module in the current study - module 15). Finally, there are an additional 25 proteins representing a variety of functions including minD (which we group into module 132 together with other cell division regulators - minC, minE and dicB); the ribosomal protein rpsB (which we group into module 8 with other translation related proteins); the cell wall synthesis protein mraY (which we group into module 9 with other cell wall proteins); and the two component system proteins - atoC and atoS (which we group into separate modules with related components). Hence, whereas the Hu et al. GC module analysis appears to group many different functions into a single heterogeneous module, the modular analysis presented here analysis is able to partition these different functions appropriately.

There are two contributing factors that likely account for these observed differences compared to our own functional modules. Firstly, the larger dataset and choice of inflation parameter has led to the merging of a variety of modules in the Hu et al. GC dataset (proteins involved in chemotaxis and flagella biosynthesis are split into two modules in our dataset). Secondly, the Hu et al. GC dataset and our own functional dataset each contain their own sets of unique interactions. The functional heterogeneity associated with the Hu et al. GC modules could therefore either reflect:

1) The lower quality of this dataset (Fig. S2). Incorrectly assigned interactions may be affecting module definition.
2) Novel roles for proteins in functional modules previously considered to be unrelated.

Investigation of these potential factors would benefit from the development and application of clustering methods, such as fuzzy clustering, that allow the partitioning of proteins into several modules.

Estimating the size of the *E. coli* interactome

A previous study by Hart and co-workers [6] used the hypergeometric distribution to estimate that the full yeast protein-protein interaction network contains from 37,800 to 75,500 interactions. We applied the same method to two independently derived experimental networks to estimate the full size of the *E. coli* interactome. Using the formula:

$N = (n_1(1\text{-}fpr_1) \times n_2\,(1\text{-}fpr_2))/k$

Where N is the estimated number of interactions, n_1 and n_2 are the number of interactions associated with the two datasets, $fpr_1$and fpr_2 are the false positive rates associated with the two datasets and k is the number of interactions common to both datasets. From the *Hu et al. TAP* dataset of 3,888 interactions, we use the 0.70 confidence score cut-off to derive a false positive rate of 0.30. The recent pull down dataset of 11,174 interactions used in our data integration [7], describes an overlap with the DIP database of ~16% from which we derive a false positive rate of 0.84. These two datasets were found to share 217 interactions. Feeding these numbers into the equation above provides a rough estimate of 22,400 total interactions. Hence we predict that our combined network, which features ~ half of the *E. coli* proteome, contains ~ one third of all *E. coli* interactions.

Network analyses in the context of COG functional categories

Focusing on the more comprehensive combined network we examined the differences between the types of interactions found between different COG functional categories. We therefore calculated a variety of graph metrics: node degree, betweenness, shortest path length, node clustering coefficient and mutual clustering coefficient – see Suppl. methods (Fig. S4). From the distributions of the graphs, distinct properties associated with individual or groups of COG categories could be discerned. While aspects of this analysis are outlined in the main paper, it is worth highlighting some additional features. Both types of clustering coefficients (node and mutual) provide a measure of the tendency of proteins to form discrete clusters within the network, however the mutual clustering coefficient additionally provides the ability to discern between forming clusters with proteins of the same or different categories. While proteins with the COG assignment N (Cell motility) had the highest clustering coefficients (Fig. S4C and D), it is interesting to note the tendency of such proteins to co-cluster with each other rather than proteins from other categories (this may be discerned by the increase in mutual cluster coefficients for proteins within the same category compared with proteins from different categories). Similar tendencies were observed for COG categories H (Coenzyme transport and metabolism) and P (Inorganic ion transport and metabolism), suggesting that proteins from these three COG categories tend to form discrete functional clusters among themselves. Betweenness and shortest path length provide measures of how central a protein is in a network, proteins possessing higher betweenness and lowest shortest path length tending to be located at the centre of a network. As noted in the main text, proteins in COG categories J (Translation, ribosomal structure and biogenesis) and L (Replication, recombination and repair) tend to be more centrally located in the network (Fig. S4A and B). However additionally a substantial fraction of proteins in COG category H (coenzyme transport and metabolism) are also centrally placed in the network, perhaps reflecting the varied biological processes in which coenzymes partake. On the other hand, proteins assigned to categories F (nucleotide transport and metabolism), P (inorganic ion transport and metabolism), Q (secondary metabolites biosynthesis, transport and catabolism), R (general function prediction only) and S (function unknown) are not well connected (low node degree) and are peripheral to the network (low betweenness values). The first three categories represent proteins involved in transport/metabolism related processes and may include non-essential proteins that either provide marginal but evolutionary important contributions to fitness or facilitate adaptation of the bacteria to changing environments [8]. The latter two categories suggest the lack of annotation associated with these genes may stem from their peripheral roles

within biological processes.

Exploring these features in more depth across all three networks (functional, *Hu et al. TAP* and combined), we examined the distribution of interactions and shortest path length between proteins for each pair of COG categories (Fig. 1E and Fig. S5). Many differences are observed between the *Hu et al. TAP* and functional network. For example in the *Hu et al. TAP* network, there was a significant enrichment in interactions between proteins in COG category J with a large number of other COG categories (I, K, L, M, O, Q, R, S, U, V and multi), such overrepresented interactions were restricted only to categories K and U in the functional network. Since similar numbers of proteins in category J were included in both networks, these findings highlight the tendency of the TAP approach to pull down additional proteins that may not directly interact with the bait protein and suggests that proteins in COG category J (which include ribosomal proteins) form large complexes that promiscuously interact with proteins from many different functional categories. In terms of clusters of categories, the functional network shows a significant enrichment in interactions between proteins derived from COG categories D (Cell cycle control, cell division, chromosome partitioning), M (Cell wall/membrane/envelope biogenesis) and U (Intracellular trafficking, secretion and vesicular transport) while the *Hu et al. TAP* network shows a significant enrichment in interactions between proteins derived from COG categories D, U and O (posttranslational modification, protein turnover, chaperones).
The biological significance of the combined interactions between COG categories D, M O and U has already been commented in the main text, however the failure of the *Hu et al. TAP* network to find enrichment for COG category M within this group likely stems from the smaller number of proteins from this category identified in the TAP assays. On the other hand, the failure of the functional network to identify enrichment for COG category O within this group may again be due to the TAP approach to identify many promiscuous interactions through large complex interactions.

Prediction of functional annotations for unknown genes

A major goal for many functional genomics and proteomics projects is the generation of accurate functional information for every gene and its product. Although tremendous progress has been made through the application of such systematic studies, we note that within the *E. coli* proteome 637 (15%) proteins have not been assigned a functional category according to the latest release of COG, while 316 (7.6%) have been assigned category S ('function unknown) and a further 340 proteins have only been assigned into category 'R' ('general function prediction only'). Recently there has been much progress in the development of novel methods of functional inference based on network connectivity [9,10]. The availability of a large scale reliable network of functional interactions for *E. coli* thus provides a valuable resource for future studies aimed at predicting the functions of these unknowns. As an initial test of the ability of the Bayesian-derived functional network to accurately infer functional annotations we investigated two basic network-based approaches: one based on direct neighbour interactions and one based on membership within predicted functional modules. To provide estimates of the accuracy of these two approaches (and indirectly the ability of our functional network to infer reliable annotations), we applied a cross validation procedure to re-predict functional annotations of proteins within the functional network (see Suppl. methods). Applying the neighbour linkage approach, we were able to identify correct annotations for 66% and 75% of the proteins depending on COG category

assignment stringency (Fig. S6C). On the other hand, applying the functional module approach led to correct annotation identification for 74% and 100% of the proteins using equivalent levels of stringency. These results demonstrate the improved performance of the functional module over the neighbour linkage method in terms of accuracy. However, in terms of coverage, the functional module approach only provides correct annotations for 889 (34% of the COG annotated *E. coli* proteome - 2,587 proteins with meaningful COGs) and 1,093 proteins (42%), using the high and low stringency approaches respectively. This compares with 1,013 (39%) and 1,180 proteins (46%) obtained by applying the neighbour linkage approach. Applying the neighbour linkage approach to random networks highlights the importance of using high confident PPI networks to make accurate functional predictions using neighbour linkage and/or functional module approaches (Fig. S6C). In summary, the functional module approach performed better in terms of accuracy but had a lower coverage compared to the neighbour linkage approach. Interestingly, both approaches appear to be complementary since merging the results of both approaches (functional module and neighbour linkage) provides correct annotations for 1,152 (45% of the COG annotated *E. coli* proteome) and 1,376 proteins (53%) using the more and less stringent approaches respectively.

Identification of Modules of Essential Proteins

In addition to proteins from the same gene family being closer in the network (Fig. S9A), we also found that essential proteins tend to be in closer proximity than non-essential proteins (mean shortest path length of essential proteins to other essential proteins was five compared with seven for non-essential proteins to other non-essential proteins). This suggests that groups of essential proteins might form distinct functional modules [11]. From our set of 316 predicted functional modules, 42 were identified as being significantly enriched in essential proteins and are likely mediate core biological processes (Table S6). For example, fourteen modules represent the largest detected functional modules including the 30S and 50S ribosomal subunits and RNA and DNA polymerases.

Conservation of the functional network within the proteobacteria

Comparisons of proteins in the functional network to other proteobacterial genomes found that 3,546 (of 3,989 - 89%) *E. coli* interactions are potentially retained across any of 37 gammaproteobacterial genomes analysed in this study, 586 (14.7%) are retained across all gammaproteobacterial genomes, 200 (5%) are found in at least one other gammaproteobacteria but not in other taxa, and interestingly only five interactions represented by nine proteins (trpE trpL; pheS pheM; rfaK rfaZ, rfaB rfaS; rfaS rfaP) are exclusive to *E. coli.* Three other species of *E. coli* were included in the analysis (see Suppl. methods) and are therefore expected to share a large number of interactions with our functional network. Interestingly, we noted a deficit of 215 (5.4%), 240 (6%), and 347 (8.7%) potential interactions from the *E. coli* strains RIM, EDL, and CFT, respectively, compared to *E. coli* K-12. These results highlight the differences even between strains from the same species.

METHODS

Data sets for Bayesian integration

Experimental protein-protein interaction (PPI) data

Experimental PPIs from various large- and small-scale experiments in *E. coli* were collected from the Database of Interacting Proteins (DIP - downloaded November 2006) [12]. PPIs from DIP were divided into two main categories small-scale experiments and large-scale TAP assays. A third large-scale PPI data set was obtained from a recent large scale pull down study based on the use of His-tags [7].

Computational predictions

Conserved coexpression data

Conserved coexpression data have previously been used to infer functional linkages, we therefore extracted the relevant data for pairs of *E. coli* genes from a study examining coexpression patterns across five other organisms: *S, cerevisiae, Caenorhabditis elegans, Arabadopsis thaliana*, *Drosophila menaogaster* and human [13].

Genome context data

Genome context data were obtained from the Prolinks database [14] which contains information on genome context methods used to predict functional linkages between proteins. These genome context methods include: the phylogenetic profile method - which uses the presence and absence of proteins across multiple genomes; the gene cluster method - which uses genome proximity; Rosetta stone - which uses a gene fusion event in a second organism; and the gene neighbour method - which uses both gene proximity and phylogenetic distribution. We used all medium-to-high confidence functional linkages provided by Prolinks from the *E. coli* data set. All the genome context methods obtained from this combined set were initially considered as independent data sets. However, due to the relatedness of gene neighbour and gene cluster methods, these two data sets were ultimately combined into a single non-redundant – gene proximity – data set for derivation of the final functional network. The confidence scores associated to each functional linkage provided by Prolinks were not taking into account in this study.

Interologs data

The Interologs approach [15] was performed by applying BLAST [16] to the *E. coli* proteome as query versus the *Helicobacter pylori* proteome as database. Then we calculated *E. coli* orthologs (defined by BLAST best reciprocal hits with a cut-off e-value <= 10^{-10}) and mapped the *H. pylori* interactome [17] to derive *E. coli* interologs.

Literature-mining data

Literature-mined PPIs (co-citations) from *E. coli* were obtained by automatically querying the Information Hyperlinked Over Proteins database (iHOP) [18].

Benchmark set

Four different benchmark sets were used in these analyses: EcoCyC [19], the Clusters of Orthologous Group (COG) [20], the Kyoto-based KEGG [21], and the Gene Ontology (GO) annotation database [22]. EcoCyC organizes genes into four main functional categories: metabolic pathways, protein complexes, pairs of genes, A and B, where the product of gene A is a component of a transcription factor that regulates gene B, and pairs of genes, A and B, where the product of gene A is a component of a transporter of a substrate that the product of gene B

catabolizes. COG assigns genes to functions within 22 broad categories, KEGG uses 230 different functional categories at the third level of hierarchy. EcoCyc provides a total of 565 distinct terms among four main categories, and therefore provides a relatively small background probability of matching biological processes at random compared with COG and KEGG datasets. GO "biological process" annotation contains up to 16 different levels of hierarchy. In the case of GO "biological process" annotations, initial tests revealed the best performance at the ninth level of the hierarchy (data not shown). This provides a total of 1,958 distinct GO terms. For each benchmark database, functional linkages were considered to be correct if both proteins share the same functional categories.

Detection of functional modules

We identified highly connected functional modules operating within our final confident network by using the Markov cluster (MCL) algorithm [23]. MCL simulates random walks within graphs using the language of Markov (stochastic) matrices to partition a graph into highly connected modules. This procedure works efficiently on large dense graphs [24]. Furthermore, MCL algorithm was found to be remarkably robust to graph alterations and it has the best performance over other clustering algorithms on both simulated and real data sets [25]. MCL was applied to our networks by testing several inflation operators, and settling on values that provided the best overlap (semantic similarity) [26] of the computed clusters with the functional categories of the highly curated database EcoCyc [19]. All three networks (functional, *Hu et al. TAP* and combined) produced similar distributions of module sizes that were markedly different from those produced by random networks, again highlighting the non-random organization of proteins into modules (Fig. S6B).

Generation of random networks

To act as controls, three types of randomly generated networks were created. *Random networks* were created by randomly selecting equivalent numbers of proteins (compared with the comparator network) from the *E. coli* proteome and randomly connecting them with equivalent numbers of interactions. *Shuffled networks* were generated by using the same set of proteins as the comparator network and randomly assigning equivalent numbers of interactions. *Random networks:same topology* were generated using the same set of proteins as the comparator network by randomly reassigning their interaction partners, while at the same time maintaining their number of interactions within the network (i.e. the degree distribution of the network remains the same) [27].

Functional prediction

Functional predictions were performed using two methods: 1) a neighbour linkage approach [28]; and 2) an enrichment of COG terms for predicted functional modules. *Neighbour linkage* prediction was based on the COG functional annotation of the direct neighbours of the target protein. *Functional module* prediction for a protein employed the predicted functional modules and derived COG annotations for the target proteins based on the highest percentage of common COG terms among the different members of the functional module. For both methods, correct COG assignment additionally required at least 20% of the interaction partners/module members to have the same COG category. Finally two measures of stringency were employed: high stringency predictions required the majority of interaction partners/module members to be assigned to the same COG category; low stringency predictions only required any of the

interaction partners/module members to possess the same COG category (albeit with the additional proviso that at least 20% of the interactions/module partners were so annotated). To measure the accuracy of functional predictions, we used the leave-one-out cross validation (LOOCV) procedure, i.e. only proteins which itself and one of its neighbours possessed an annotation were used in cross validation. The LOOCV method randomly selects a protein and compares its known function with that predicted by the neighbourhood linkage or functional module methods.

Network analyses

Unless otherwise noted network analyses were performed using Perl scripts developed in house. Values of Betweenness and network diameter were obtained using Pajek [29]. Node cluster coefficients were obtained using tYNA [30].

Node degree

The degree (*k*) of a node (protein) in a PPI network is defined by the number of interactions of the node with other nodes in the network.

Mutual clustering coefficient

The cumulative hypergeometric distribution is frequently used to measure cluster enrichment and significance of co-occurrence. The summation in the hypergeometric coefficient can be interpreted as a p-value, the probability of obtaining a number of mutual neighbours between two nodes at or above the observed number by chance, under the null hypothesis that the neighbourhoods are independent, and given both the neighbourhood sizes of the two nodes and total number of nodes [31]. For two proteins P_i and P_j, let d_i and d_j denote the number of interactors of P_i and P_j, respectively, d_{ij} stands for the number of common interactors of P_i and P_j. Therefore, the hypergeometric mutual clustering coefficient of P_i and P_j ($Hypergeo_{ij}$) is defined as:

$$Hypergeo_{ij} = -\log \sum_{k=d_{ij}}^{\min(d_i, d_j)} \frac{\binom{d_i}{k} \times \binom{N-d_i}{d_j-k}}{\binom{N}{d_j}}$$

where N stands for the total number of proteins.

Shortest path length

The shortest path length between two nodes in the network is the number of edges in a shortest path connecting them. The shortest path length is infinity if there are no paths between two nodes. We use the Dijkstra's algorithm [32] to calculate the shortest path length between every two nodes in the network.

Eccentricity

The eccentricity of a node P_i is the greatest distance between the node P_i and any other nodes [32].

SUPPLEMENTAL REFERENCES (also for Figs. S1-S9)

1. Hakes L, Pinney JW, Robertson DL, Lovell SC (2008) Protein-protein interaction networks and biology-what's the connection? Nat Biotech 26: 69-72.
2. Yellaboina S, Goyal K, Mande SC (2007) Inferring genome-wide functional linkages in E. coli by combining improved genome context methods: comparison with high-throughput experimental data. Genome Res 17: 527-535.
3. von Mering C, Huynen M, Jaeggi D, Schmidt S, Bork P, et al. (2003) STRING: a database of predicted functional associations between proteins. Nucleic Acids Res 31: 258-261.
4. von Mering C, Jensen LJ, Kuhn M, Chaffron S, Doerks T, et al. (2007) STRING 7--recent developments in the integration and prediction of protein interactions. Nucleic Acids Res 35: D358-362.
5. Hu P, Janga SC, Babu M, Diaz-Mejia JJ, Butland G, et al. (2009) Global functional atlas of Escherichia coli encompassing previously uncharacterized proteins. PLoS Biol 7: e96.
6. Hart GT, Ramani AK, Marcotte EM (2006) How complete are current yeast and human protein-interaction networks? Genome Biol 7: 120.
7. Arifuzzaman M, Maeda M, Itoh A, Nishikata K, Takita C, et al. (2006) Large-scale identification of protein-protein interaction of Escherichia coli K-12. Genome Res 16: 686-691.
8. Thatcher JW, Shaw JM, Dickinson WJ (1998) Marginal fitness contributions of nonessential genes in yeast. Proc Natl Acad Sci U S A 95: 253-257.
9. McDermott J, Bumgarner R, Samudrala R (2005) Functional annotation from predicted protein interaction networks. Bioinformatics 21: 3217-3226.
10. Vazquez A, Flammini A, Maritan A, Vespignani A (2003) Global protein function prediction from protein-protein interaction networks. Nat Biotechnol 21: 697-700.
11. Reguly T, Breitkreutz A, Boucher L, Breitkreutz BJ, Hon GC, et al. (2006) Comprehensive curation and analysis of global interaction networks in Saccharomyces cerevisiae. J Biol 5: 11.
12. Salwinski L, Miller CS, Smith AJ, Pettit FK, Bowie JU, et al. (2004) The Database of Interacting Proteins: 2004 update. Nucleic Acids Res 32: D449-451.
13. Bergmann S, Ihmels J, Barkai N (2004) Similarities and differences in genome-wide expression data of six organisms. PLoS Biol 2: E9.
14. Bowers PM, Pellegrini M, Thompson MJ, Fierro J, Yeates TO, et al. (2004) Prolinks: a database of protein functional linkages derived from coevolution. Genome Biol 5: R35.
15. Yu H, Luscombe NM, Lu HX, Zhu X, Xia Y, et al. (2004) Annotation transfer between genomes: protein-protein interologs and protein-DNA regulogs. Genome Res 14: 1107-1118.
16. Altschul SF, Gish W, Miller W, Myers EW, Lipman DJ (1990) Basic local alignment search tool. J Mol Biol 215: 403-410.
17. Rain JC, Selig L, De Reuse H, Battaglia V, Reverdy C, et al. (2001) The protein-protein interaction map of Helicobacter pylori. Nature 409: 211-215.
18. Hoffmann R, Valencia A (2005) Implementing the iHOP concept for navigation of biomedical literature. Bioinformatics 21 Suppl 2: ii252-258.
19. Keseler IM, Collado-Vides J, Gama-Castro S, Ingraham J, Paley S, et al. (2005) EcoCyc: a comprehensive database resource for Escherichia coli. Nucleic Acids Res 33: D334-337.
20. Tatusov RL, Fedorova ND, Jackson JD, Jacobs AR, Kiryutin B, et al. (2003) The COG

database: an updated version includes eukaryotes. BMC Bioinformatics 4: 41.
21. Kanehisa M, Goto S, Hattori M, Aoki-Kinoshita KF, Itoh M, et al. (2006) From genomics to chemical genomics: new developments in KEGG. Nucleic Acids Res 34: D354-357.
22. Ashburner M, Ball CA, Blake JA, Botstein D, Butler H, et al. (2000) Gene ontology: tool for the unification of biology. The Gene Ontology Consortium. Nat Genet 25: 25-29.
23. van Dongen S (2000) Graph clustering by flow simulation: University of Utrecht.
24. Krogan NJ, Cagney G, Yu H, Zhong G, Guo X, et al. (2006) Global landscape of protein complexes in the yeast Saccharomyces cerevisiae. Nature 440: 637-643.
25. Brohee S, van Helden J (2006) Evaluation of clustering algorithms for protein-protein interaction networks. BMC Bioinformatics 7: 488.
26. Lord PW, Stevens RD, Brass A, Goble CA (2003) Investigating semantic similarity measures across the Gene Ontology: the relationship between sequence and annotation. Bioinformatics 19: 1275-1283.
27. Maslov S, Sneppen K (2002) Specificity and Stability in Topology of Protein Networks. pp. 910-913.
28. Schwikowski B, Uetz P, Fields S (2000) A network of protein-protein interactions in yeast. Nat Biotechnol 18: 1257-1261.
29. Batagelj V, Mrvar A (1998) Pajek - Program for large network analysis. Connections 21: 47-57.
30. Yip KY, Yu H, Kim PM, Schultz M, Gerstein M (2006) The tYNA platform for comparative interactomics: a web tool for managing, comparing and mining multiple networks. Bioinformatics 22: 2968-2970.
31. Goldberg DS, Roth FP (2003) Assessing experimentally derived interactions in a small world. Proc Natl Acad Sci U S A 100: 4372-4376.
32. West D (2001) Introduction to graph theory: Prentice-Hall Inc.
