# Supplementary figures and images for: The Modular Organization of Protein Interactions in Escherichia coli
Source: PLoS Comput Biol. 2009 Oct 2;5(10):e1000523. doi: 10.1371/journal.pcbi.1000523 (PMC2739439; doi:10.1371/journal.pcbi.1000523)

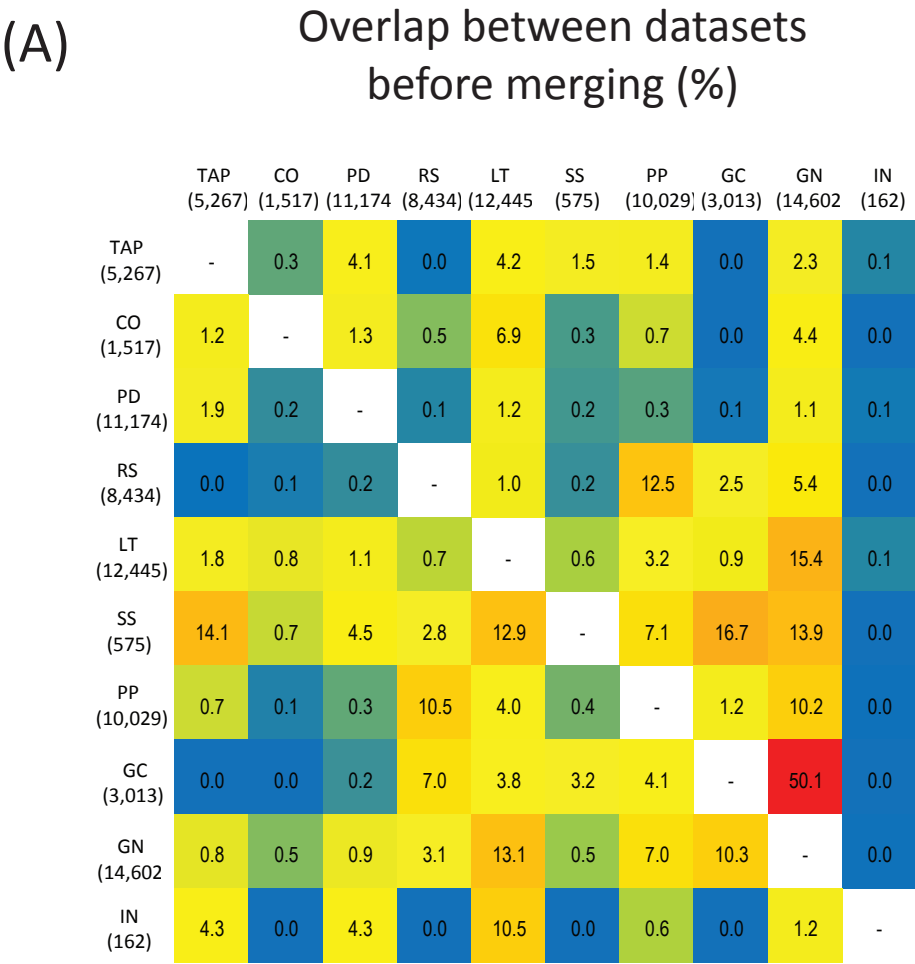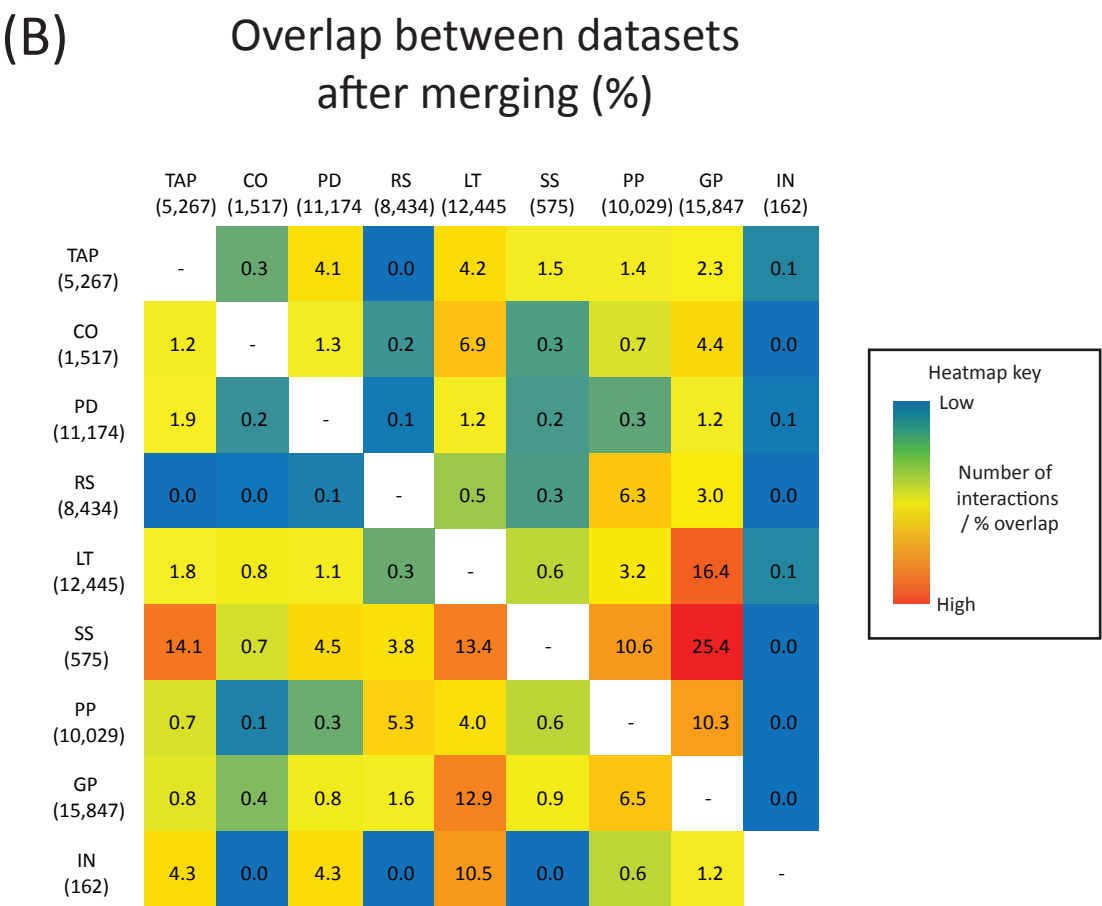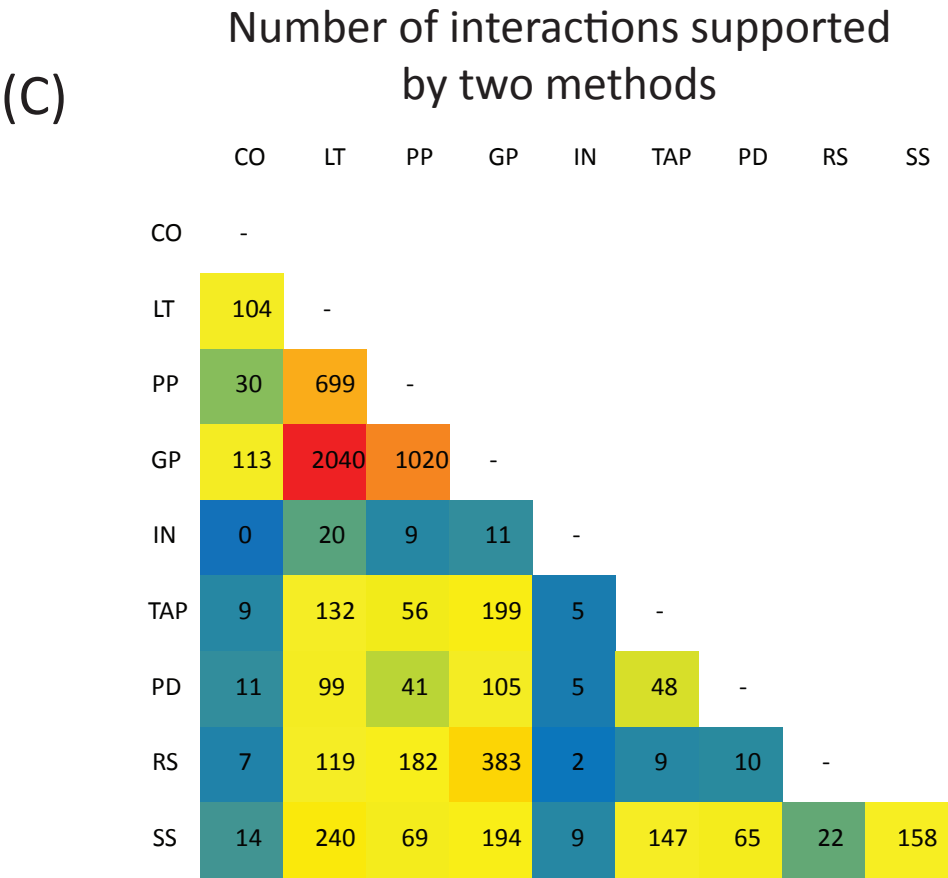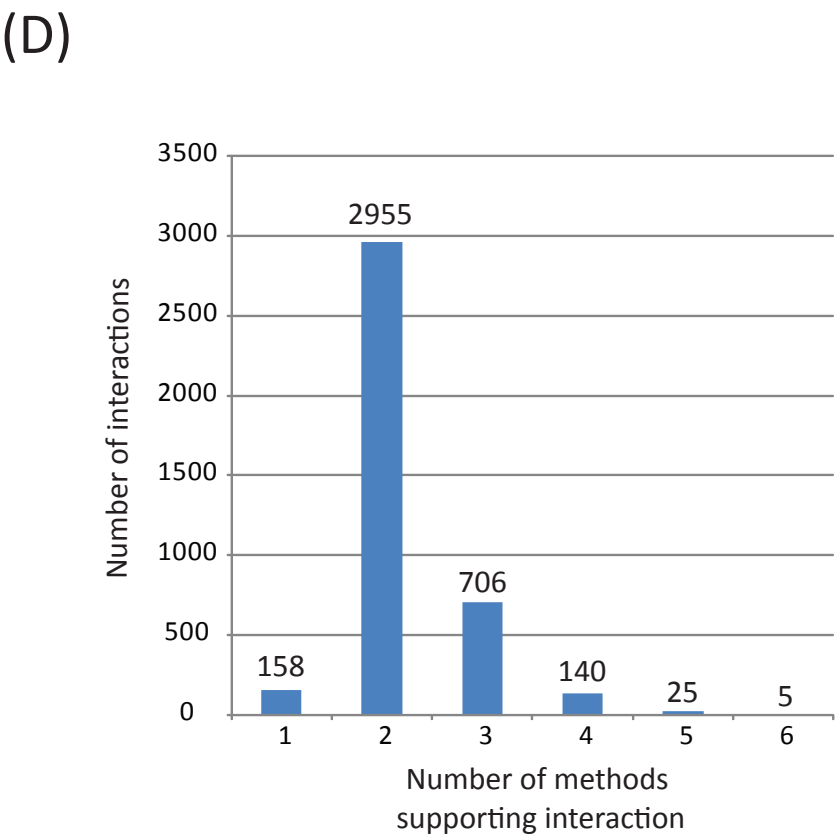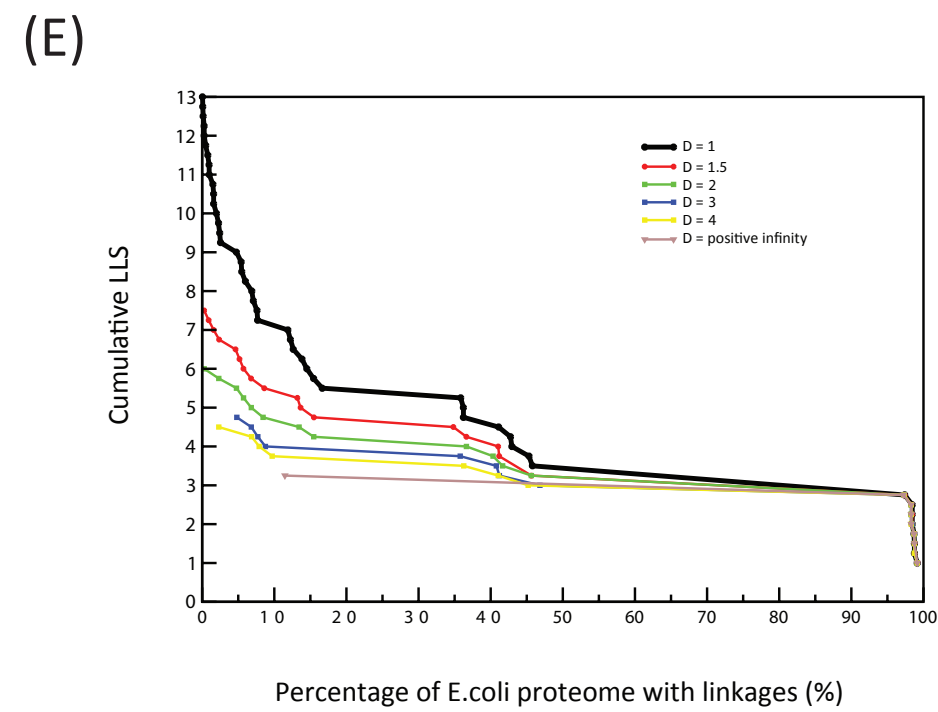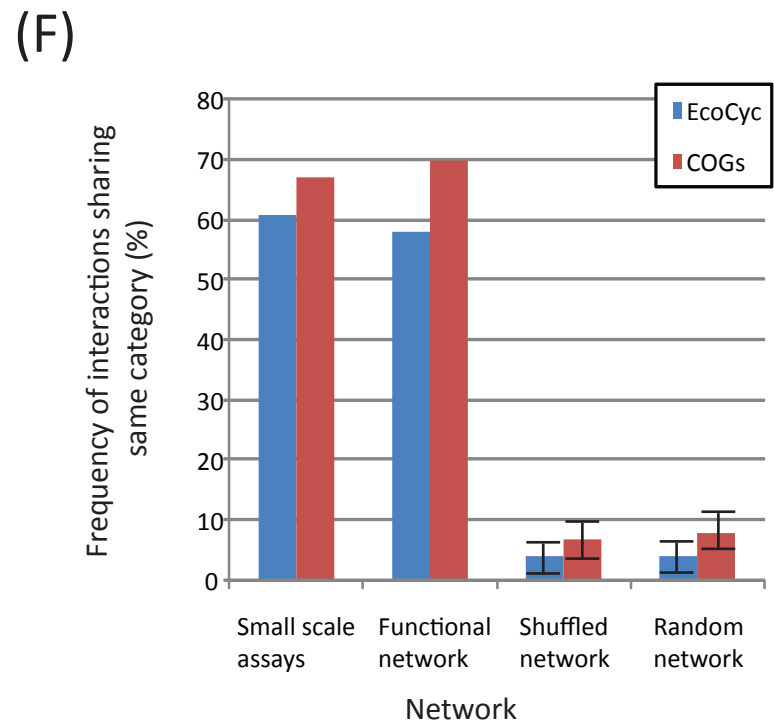

Figure S1

Supplement: Figure S1 — Dataset integration and functional overlap. (A and B) Overlap between the data sets used for inferring functional interactions. In an initial approach ten methods were integrated via a Bayesian framework to predict functional interactions. (A) shows the percentage of these interactions supported by one method that are also supported by each of the nine other methods. In the definitive approach used to derive the ‘functional network’ described here, we combined the gene cluster and gene neighbourhood data sets into a single non-redundant set (genome proximity). The data sets were then reanalyzed to predict 3989 interactions between 1941 proteins. The graphic in (B) shows the percentage of these new interactions supported by one method that are also supported by each of the other eight methods. Background colour scales from the highest level of overlap (red) to the lowest (blue). TAP = Tandem Affinity Purification; CO = Conserved Co-expression; PD = Pull Down experiments; RS = Rosetta Stone; LT = Literature mining; SS = Small Scale experiments; PP = Phylogenetic profiles; GC = Gene Cluster; GN = Gene Neighbourhood; GP = Genome Proximity (see above); and IN = Interologs (see supplemental methods for sources of data). Number of functional linkages inferred by each method is given in brackets. (C) Number of interactions in the functional network supported by one method that are also supported by each of the eight other methods. Background colour scales from the highest values (red) to the lowest (blue). (D) Breakdown of the number of methods used to support each of the 3989 interactions in the functional network. Note the 158 interactions supported by a single method were derived from the small scale assays that did not have any extra supporting evidence. (E) To examine potential biases that may arise from data dependencies we applied a weighted sum scoring scheme [33] to derive a final score S associated with each interaction (see methods in main text). D is a free para [file pcbi.1000523.s002.pdf]

(A)

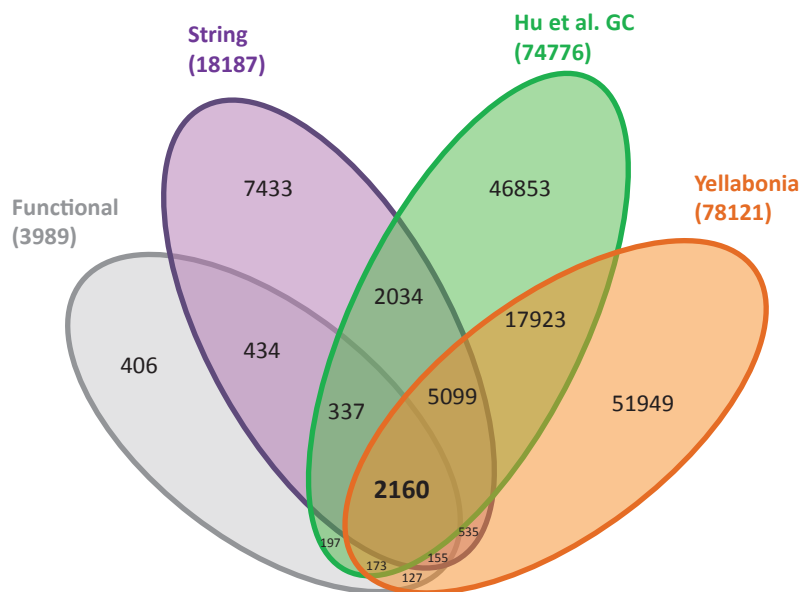

(B)

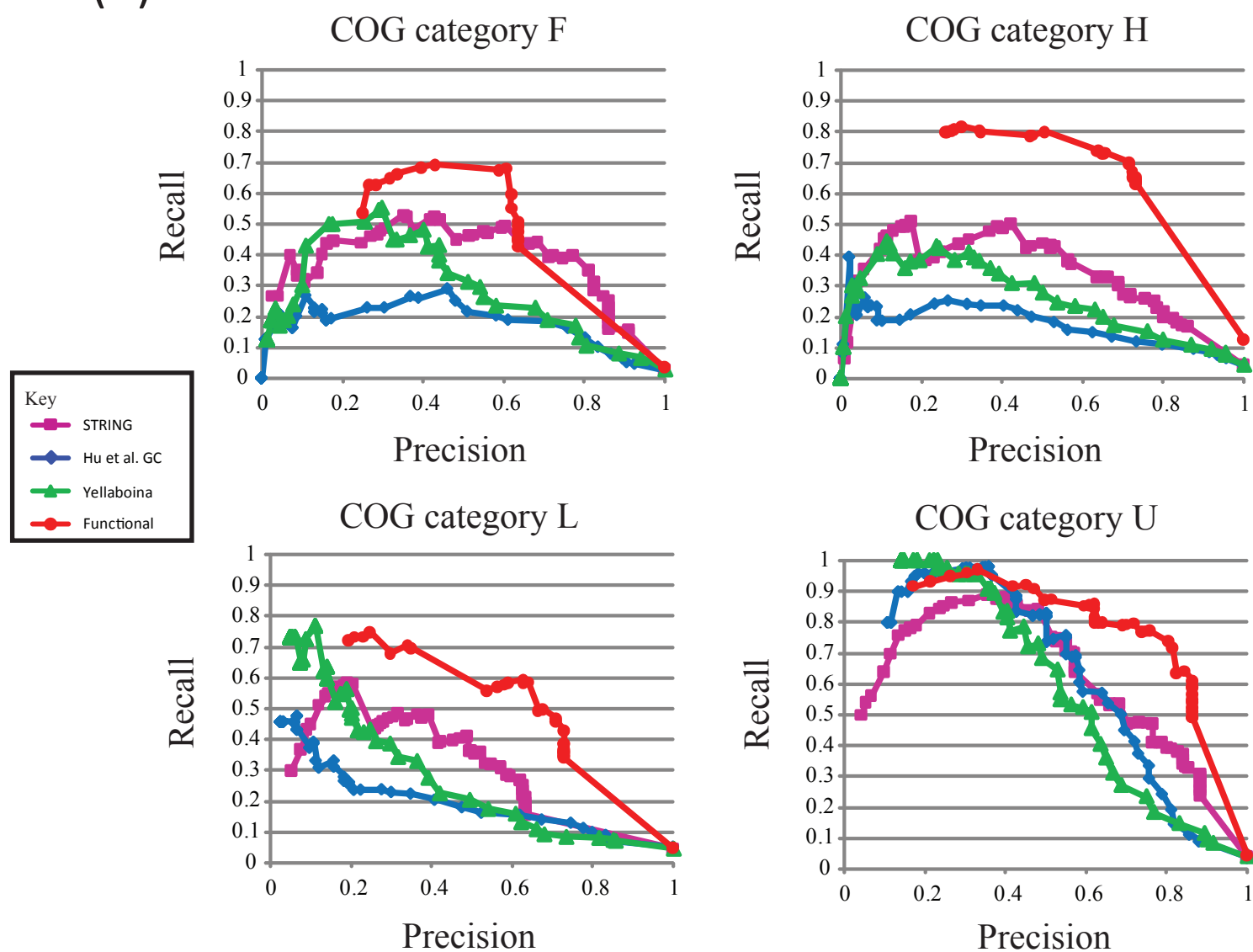

Figure S2A & B

(C)

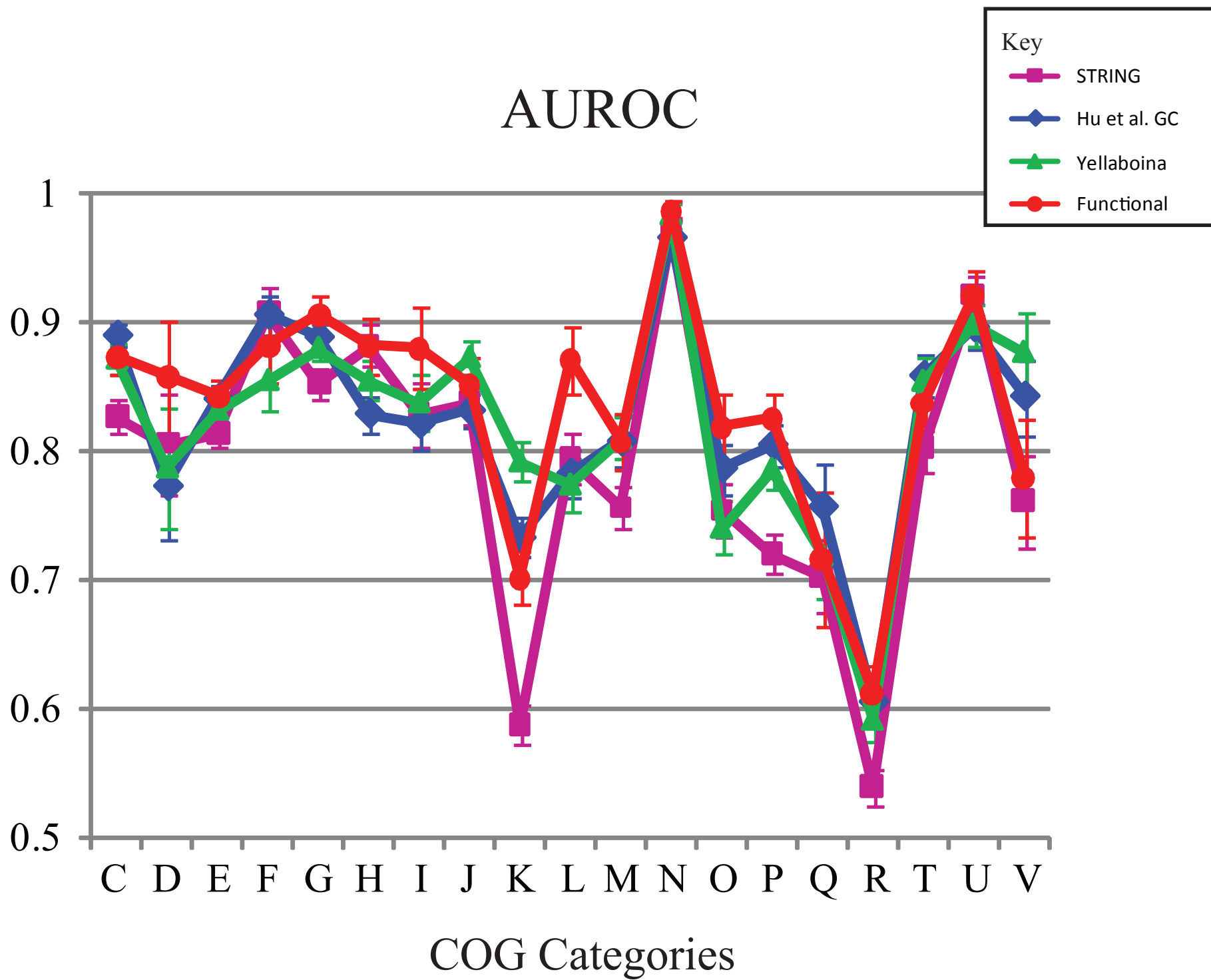

Figure S2C

(D)

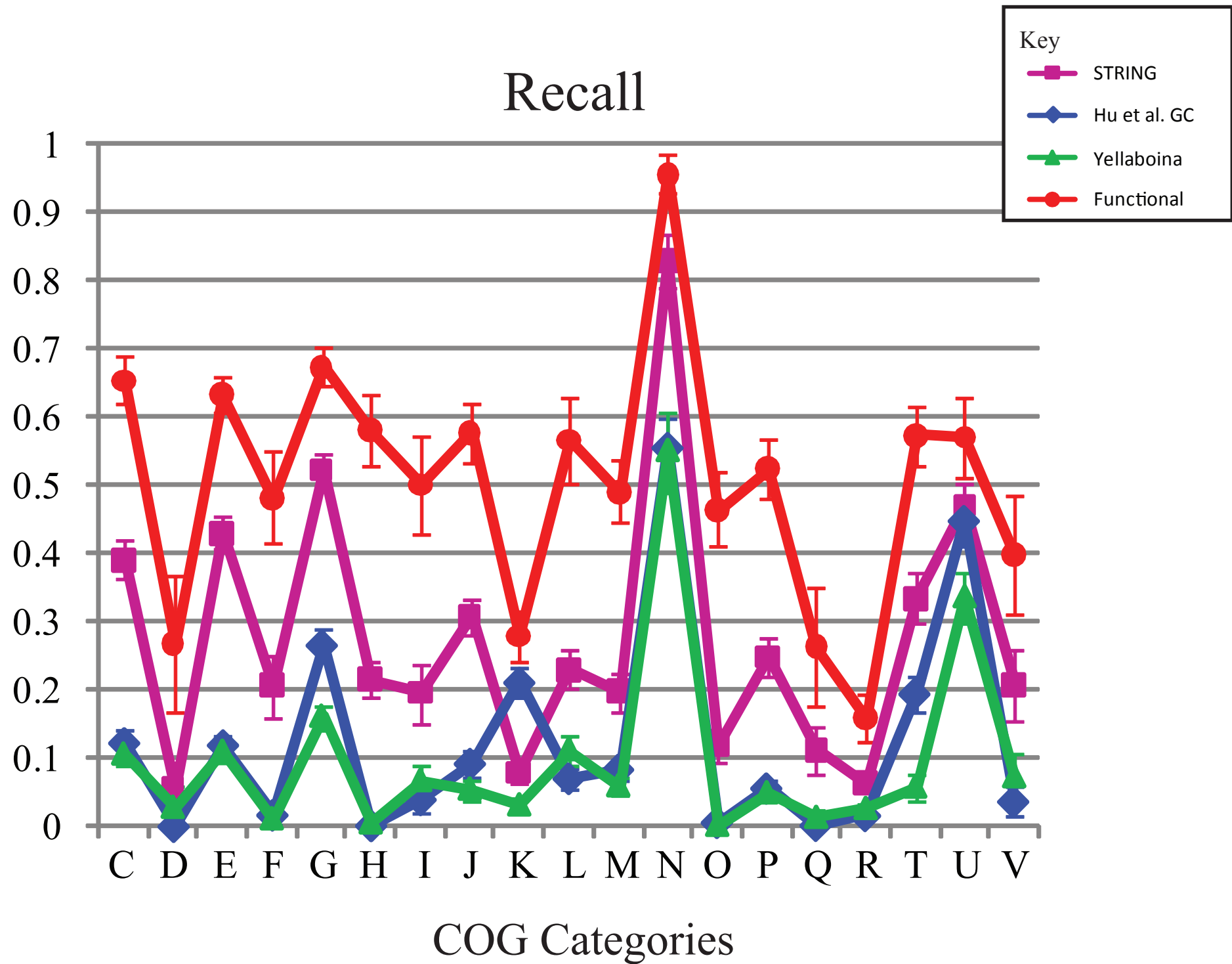

Figure S2D

(E)

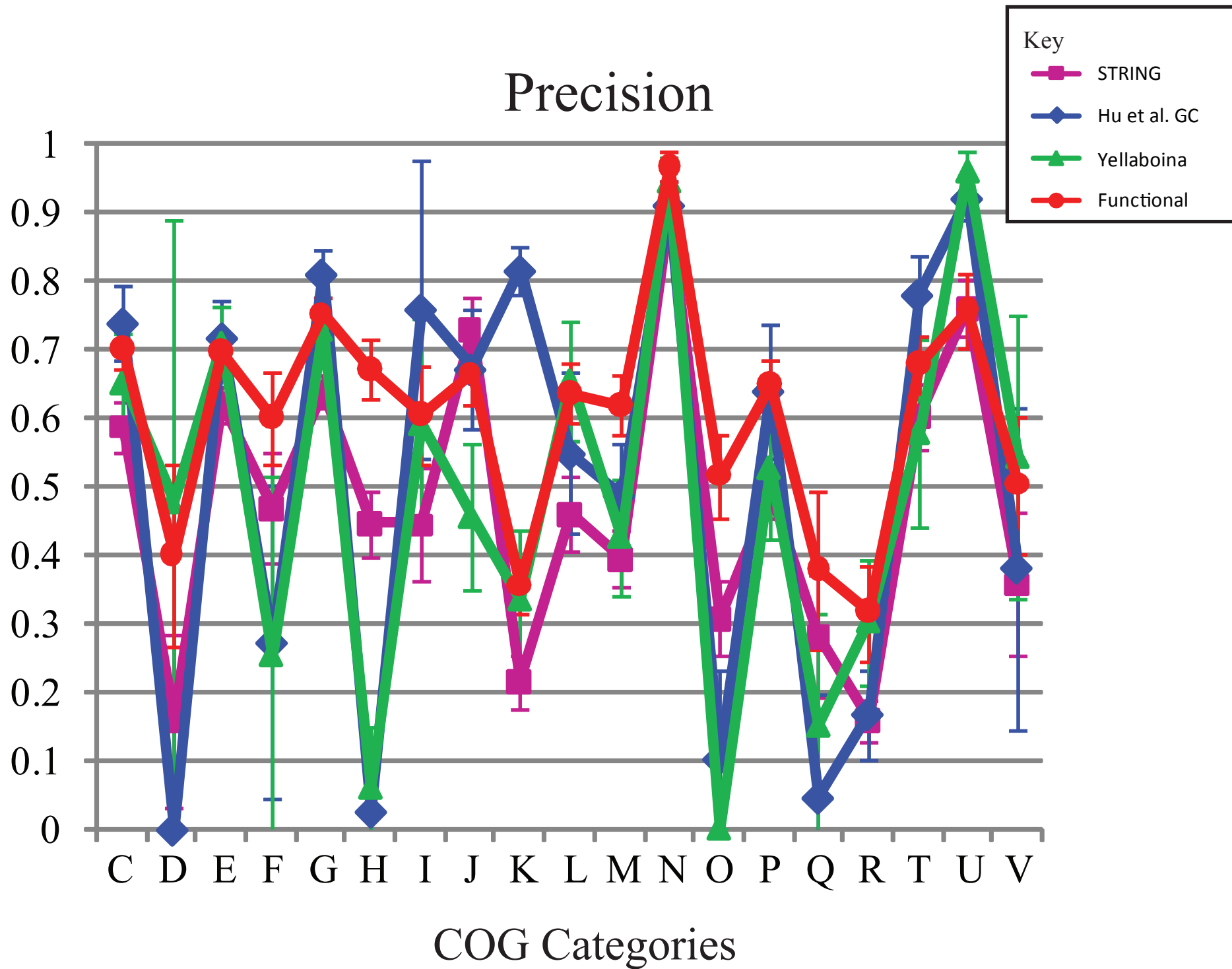

Figure S2E

Supplement: Figure S2 — Comparison of the functional network to three previously published functional networks. (A) Overlap of interactions derived from four E. coli functional interaction datasets: Functional (this study); String [4]; Hu et al., GC [5]; and Yellaboina [2]. Numbers in brackets indicate the total number of interactions associated with the dataset. (B) Sample precision-recall curves for four selected COG categories. Precision/Recall values were obtained using the five fold cross-validation method of assigning COG categories based on label propagation described in the main text. Different values of precision and recall were generated from increasing the threshold cutoff for label propagation from 0 to 1. (C–E) Measures of performance for the four functional datasets: (C) Area under the receiver operating characteristic curve (AUROC = sensitivity vs. (1-specificity)); (D) Recall; and (E) Precision. To maintain consistency across all COG categories, for graphs D and E (summarizing differences in Recall and Precision) we used a single threshold cutoff for label propagation of 0.5. Points and error bars represent the means and standard deviations obtained from 100 replicates. The functional network presented in this study significantly out performs the other three datasets in terms of improved recall across all COG categories. Furthermore, it provides the highest values of precision for 8 COG categories and the next best value of precision for an additional eight categories. Finally, in terms of AUROC values, our functional network out performs all three other datasets in 10 of 19 COG categories. (1.58 MB PDF) [file pcbi.1000523.s003.pdf]

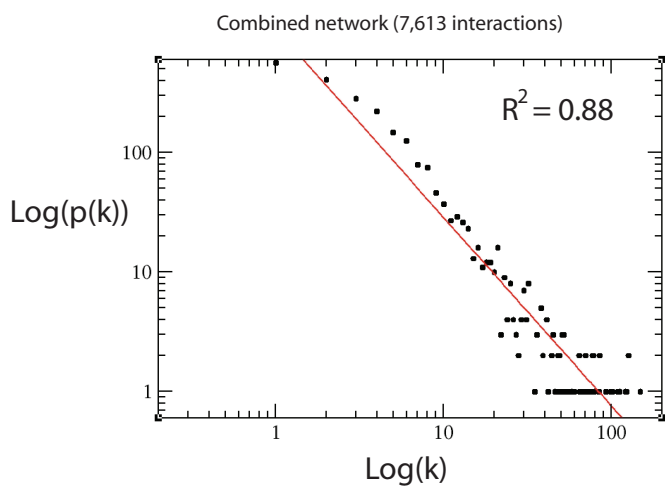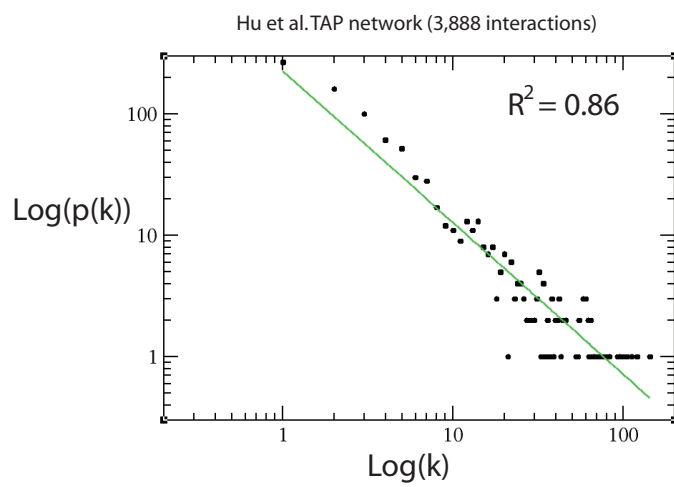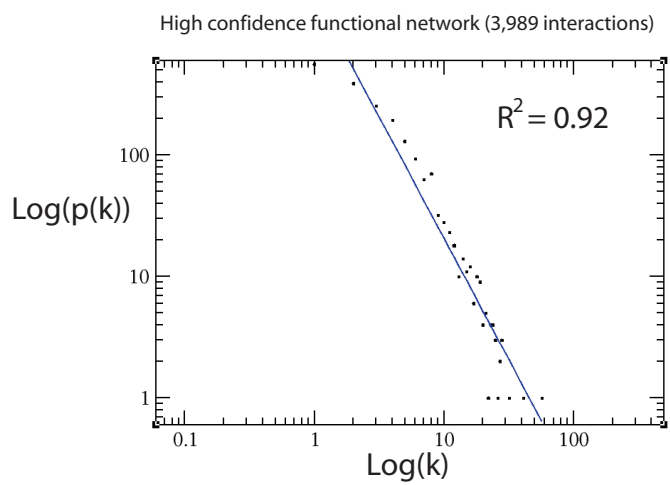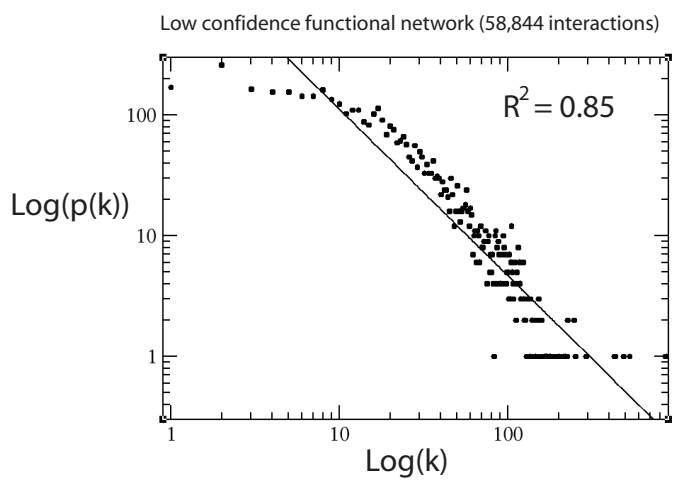

Figure S3

Supplement: Figure S3 — Scale free behavior of the networks. Node degree distributions of the four networks (combined, Hu et al. TAP, high- and low-confidence functional networks) presented in the paper. Each graph is a log-log plot of the number of interactions (k) for each protein as a function of frequency (p(k)). Each network demonstrates scale free behavior as shown by the linear relationships within each graph. (0.38 MB PDF) [file pcbi.1000523.s004.pdf]

(A)

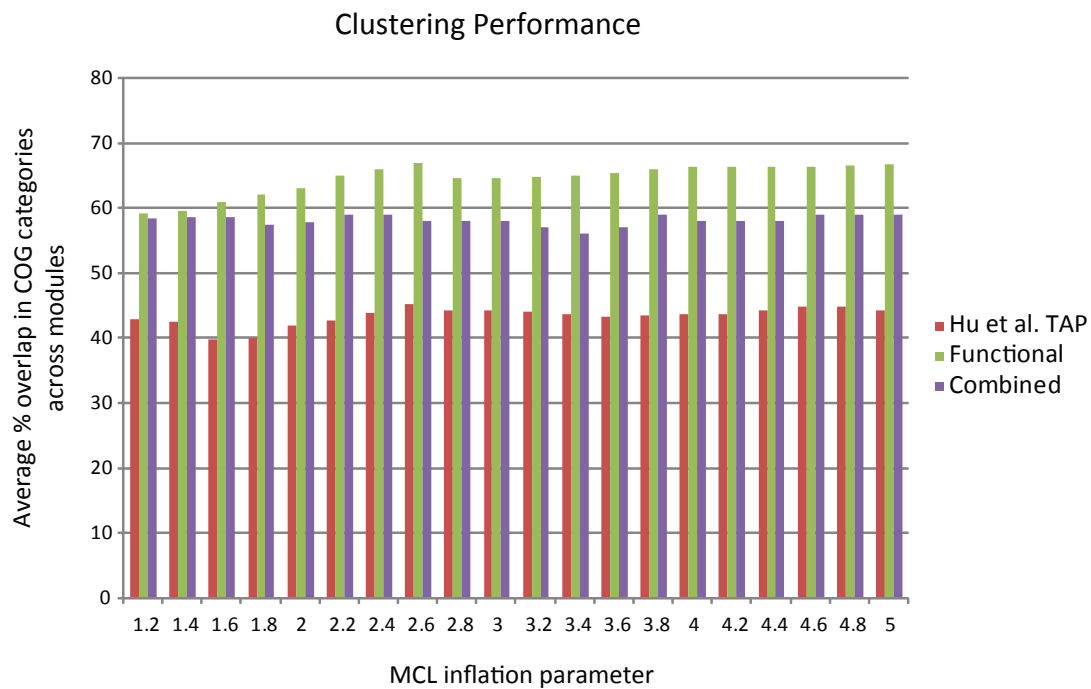

(B)

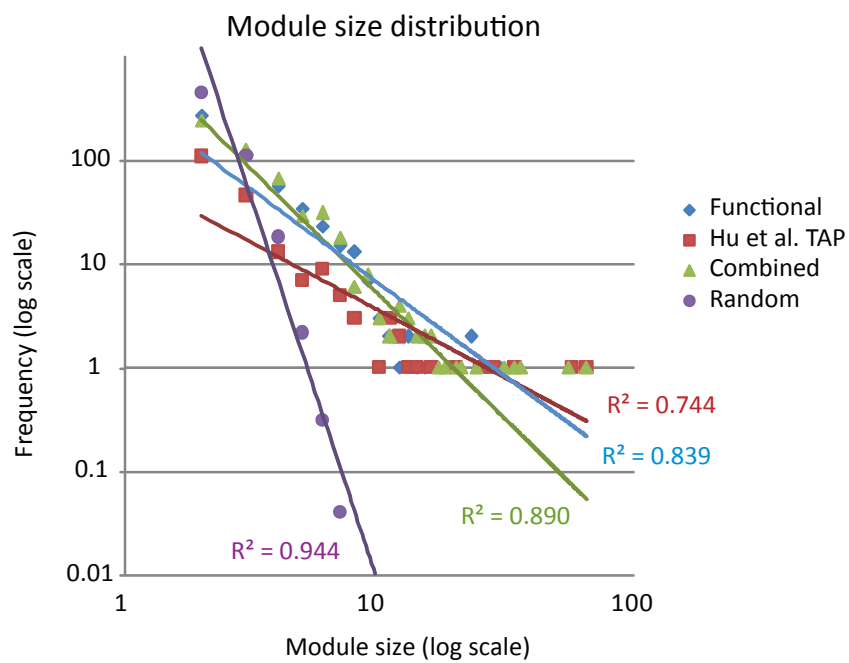

(C)

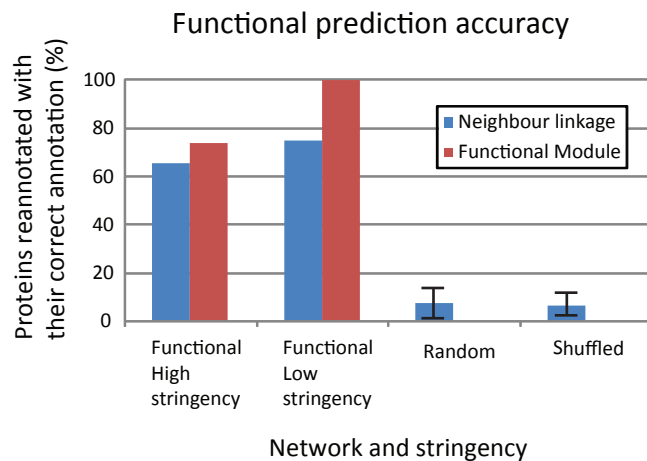

Supplement: Figure S6 — Performance of the MCL algorithm on module prediction. (A) Bars represent the average percentage of overlap in COG categories within module predictions at different MCL inflation values for the Hu et al. TAP, functional and combined networks. The percentage of overlap for each module was obtained by considering only the most abundant COG category in the module. (B) Size distribution of modules. Values represent the distribution of module sizes (log scale) for the Hu et al. TAP, functional and combined networks. The average for 100 random networks of similar size to the combined network are also shown. Lines of best fit and correlation coefficients (R-squared) are indicated for each data set. Note the relative steepness of the line associated with the random network compared with the other three networks. (C) Accuracy of functional network to predict correct COG annotations for two network-based methods: ‘neighbour linkage’ and ‘functional module’ using a leave-one-out cross-validation procedure (see Text S1 - Methods). Bars indicate the frequency of correct COG assignment. Two measures of stringency were employed: high stringency indicates that the majority of interaction partners/module members had the same COG category; low stringency indicates that any of the interaction partners/module members had the same COG category. In both cases correct COG assignment additionally required at least 20% of the interaction partners/module members to have the same COG category. Due to the difference in module size distributions (B), only the neighbour linkage method was applied to random and shuffled networks of equal size to the functional network. Error bars indicate standard deviation for 30 replicate random/shuffled controls. (0.37 MB PDF) [file pcbi.1000523.s007.pdf]

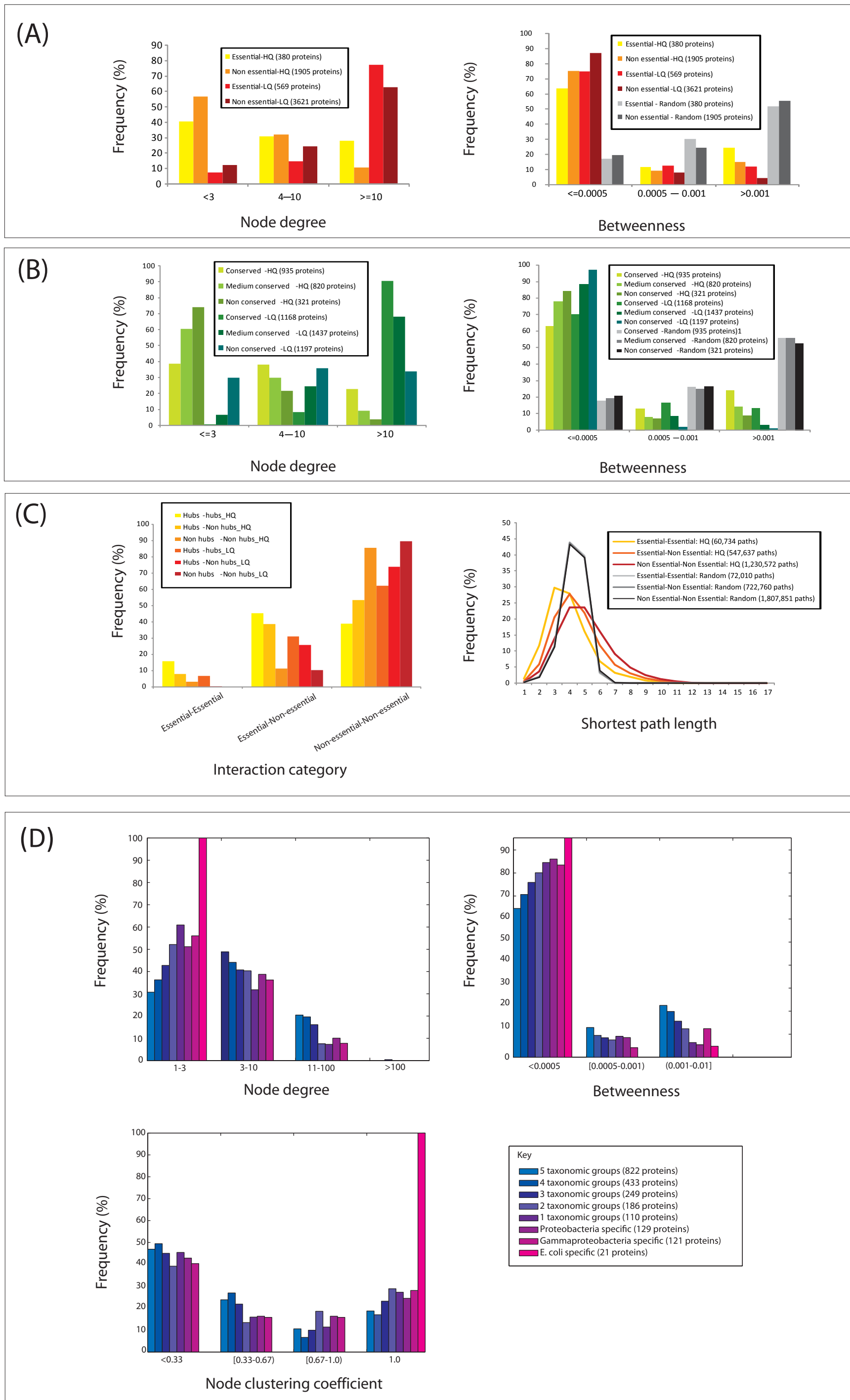

Figure S8

Supplement: Figure S8 — Network properties associated with gene conservation and essentiality. (A and B) Graphs comparing network properties (node degree and betweenness centrality) with a protein's essentiality (A) and conservation (B). Conserved proteins are defined as those with homologs in more than 100 genomes (of 199), medium conserved proteins are defined as those with homologs in 25 to 100 genomes and non conserved proteins are defined as those with homologs in less than 25 genomes. Lists of genomes are provided in Table S5. Descriptions of how network metrics are calculated are provided in Text S1 - Methods. For each graph, results are provided for both the high quality (HQ) combined network (2,283 proteins, 7,613 interactions) and a lower quality (LQ) network consisting of the extended network of functional interactions together with the Hu et al. TAP network (4,190 proteins, 60,241 interactions). The inclusion of the low quality network in these analyses which reveal similar trends to the high quality network demonstrates that our results are not influenced by the large number of false negatives associated with the high confidence network. Also shown are results for a random network constructed with the same proteins and topology as the combined network [27] (see Suppl. methods). (C) Relationship between protein essentiality, hub-non hub interactions and shortest path lengths. Hubs are defined as proteins with more than 10 interactions; non-hubs are defined as proteins with less than three interactions. Again, results for a random network constructed with the same proteins and topology as the combined network are also shown. (D) Relationship between the node degree, betweenness centrality and node clustering coefficient of a protein and its degree of conservation within prokaryotes. Each protein in the combined network is assigned one of eight conservation categories: E. coli specific; gammaproteobacterial specific; proteobacterial specific; and 1–5 other prokaryotic groups (see [file pcbi.1000523.s009.pdf]
