# Supplementary material for: The Modular Organization of Protein Interactions in Escherichia coli
Source: PLoS Comput Biol. 2009 Oct 2;5(10):e1000523. doi: 10.1371/journal.pcbi.1000523 (PMC2739439; doi:10.1371/journal.pcbi.1000523)
Supplement: Figure S4 — Distribution of topology measures for different COG functional categories within the combined network. (A) Distribution of betweenness values for each COG functional category. (B) Distribution of shortest path length between two proteins in the network calculated both for proteins from the same COG category and for proteins to other COG categories. (C) Distribution of node clustering coefficients for each COG functional category. (D) Distribution of mutual clustering coefficients for interactions involving both proteins from the same COG category and for proteins from different COG categories. (E) Distribution of node degrees for each COG category. Descriptions of COG category codes are provided in (E). (1.76 MB PDF) [file pcbi.1000523.s005.pdf]

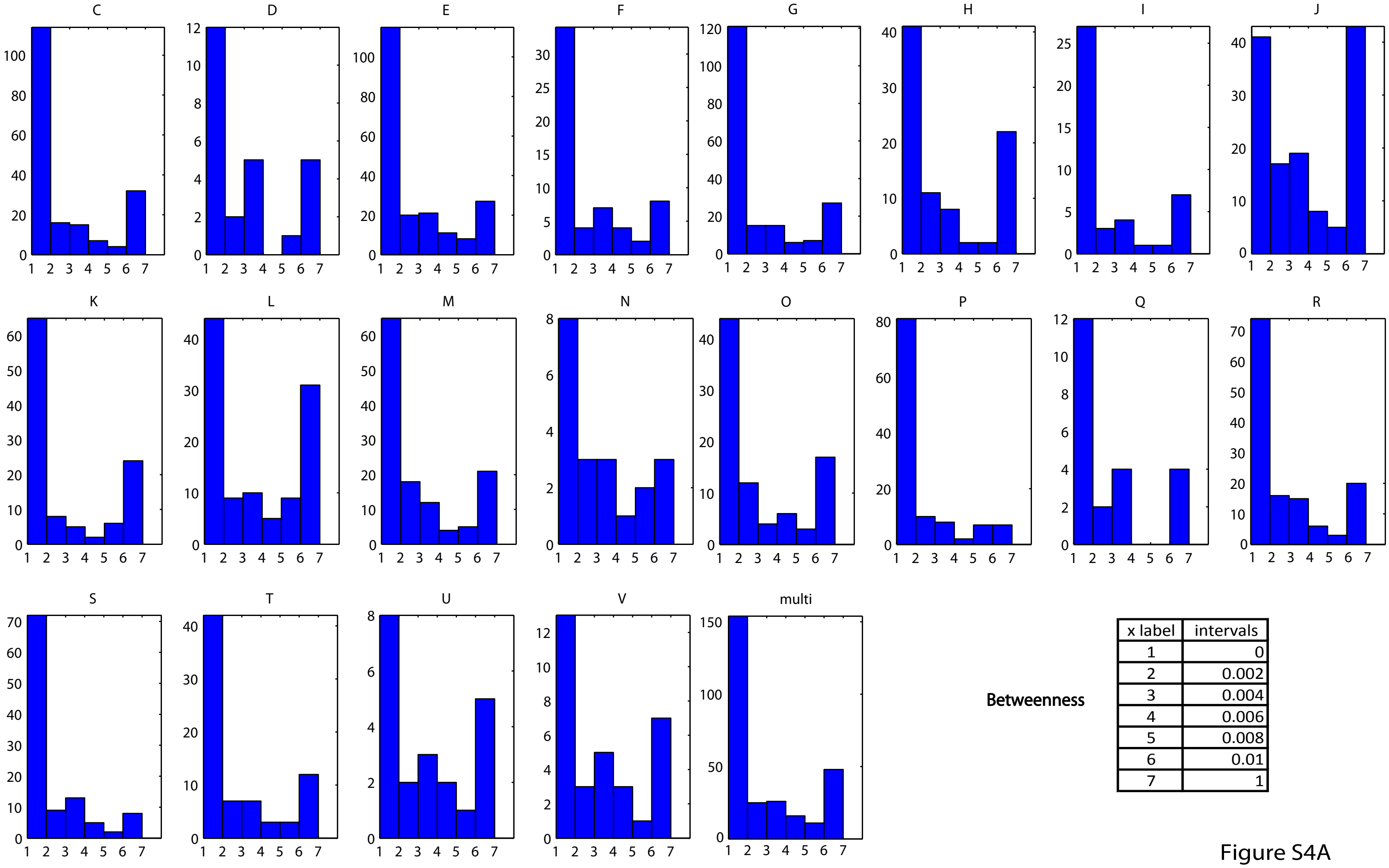

Betweenness

x label	intervals
1	0
2	0.002
3	0.004
4	0.006
5	0.008
6	0.01
7	1

Figure S4A

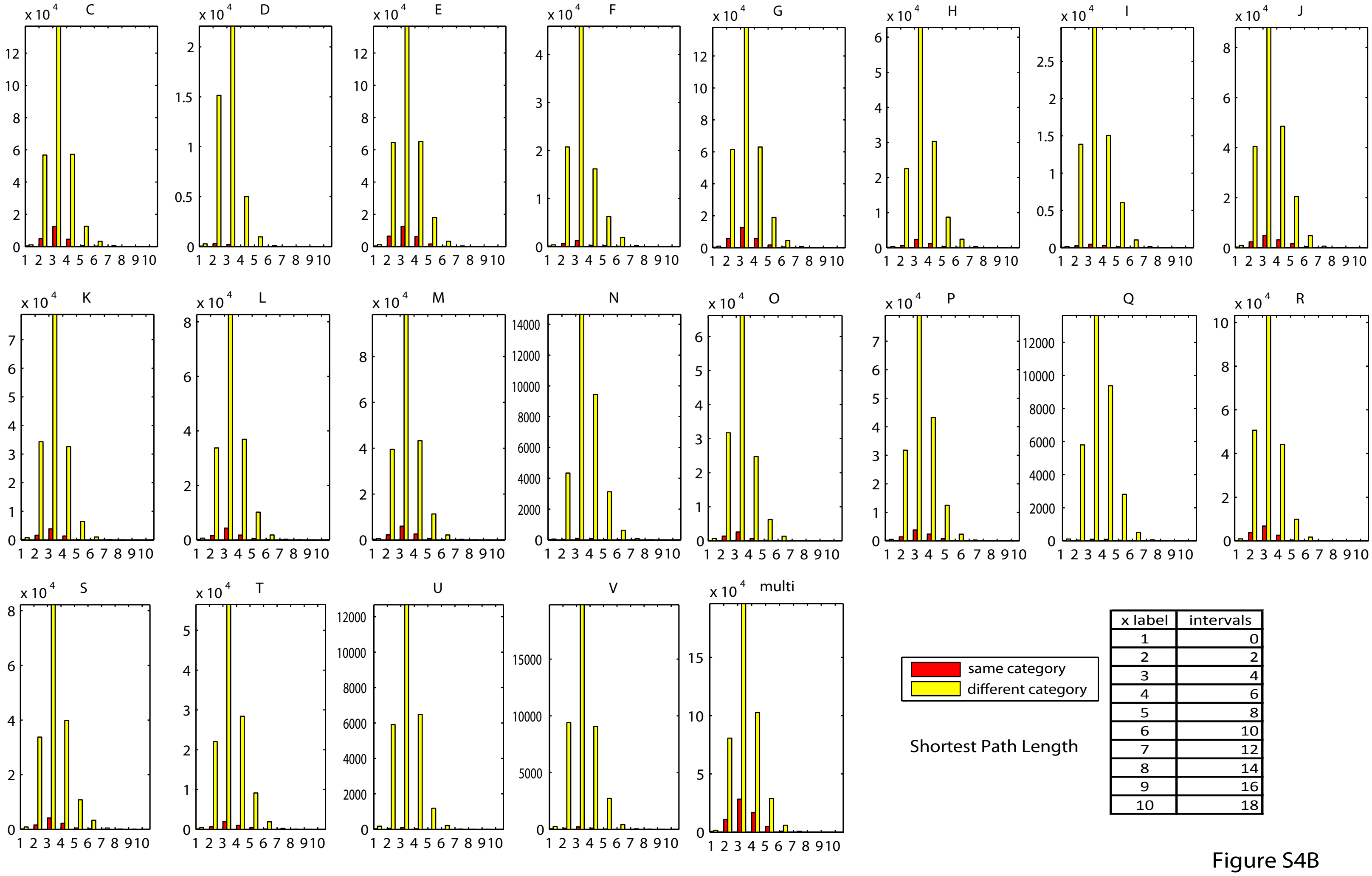

Shortest Path Length

x label	intervals
1	0
2	2
3	4
4	6
5	8
6	10
7	12
8	14
9	16
10	18

Figure S4B

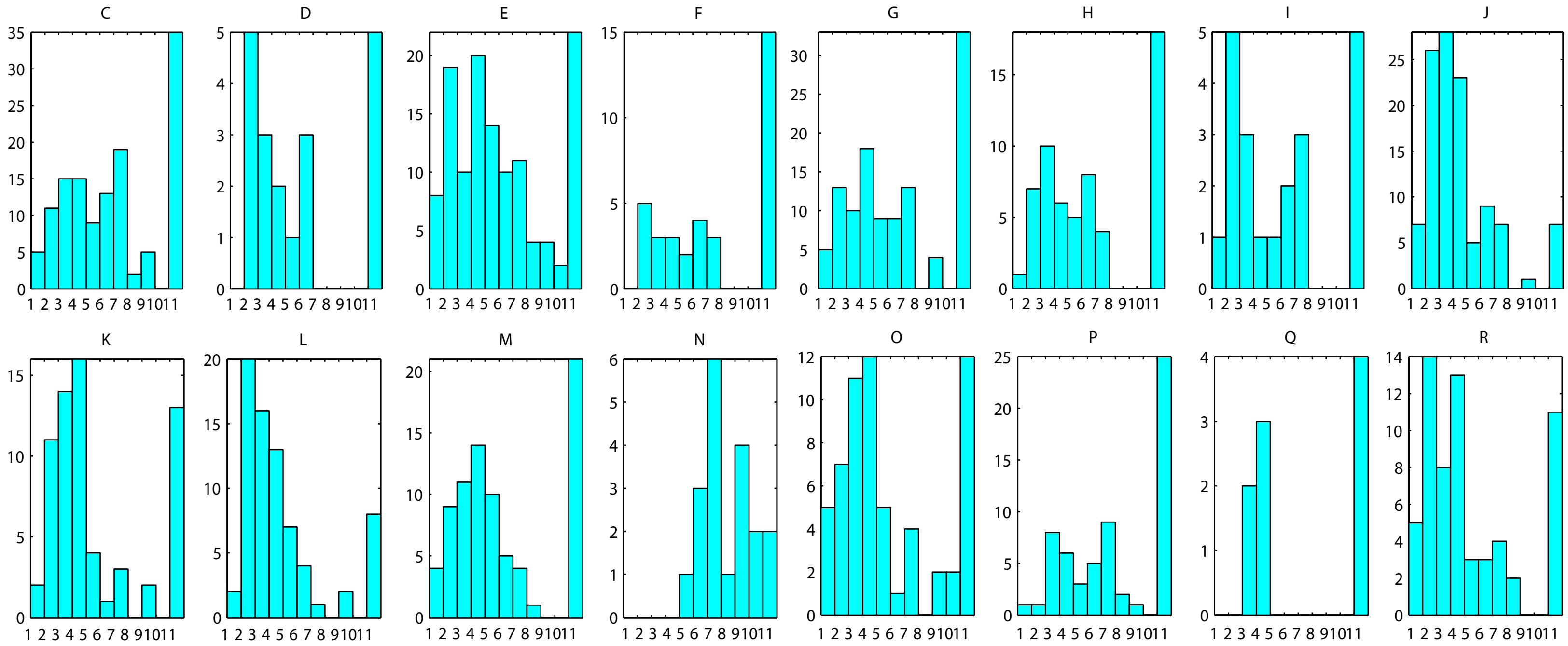

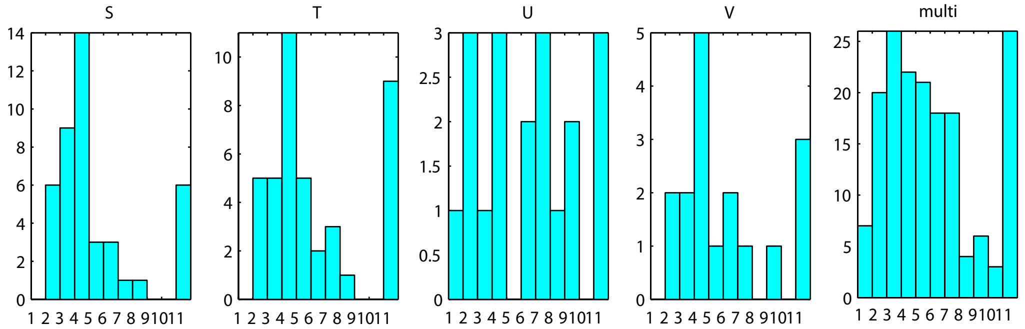

Node clustering coefficient

x label	intervals
1	0
2	0.1
3	0.2
4	0.3
5	0.4
6	0.5
7	0.6
8	0.7
9	0.8
10	0.9
11	1

Figure S4C

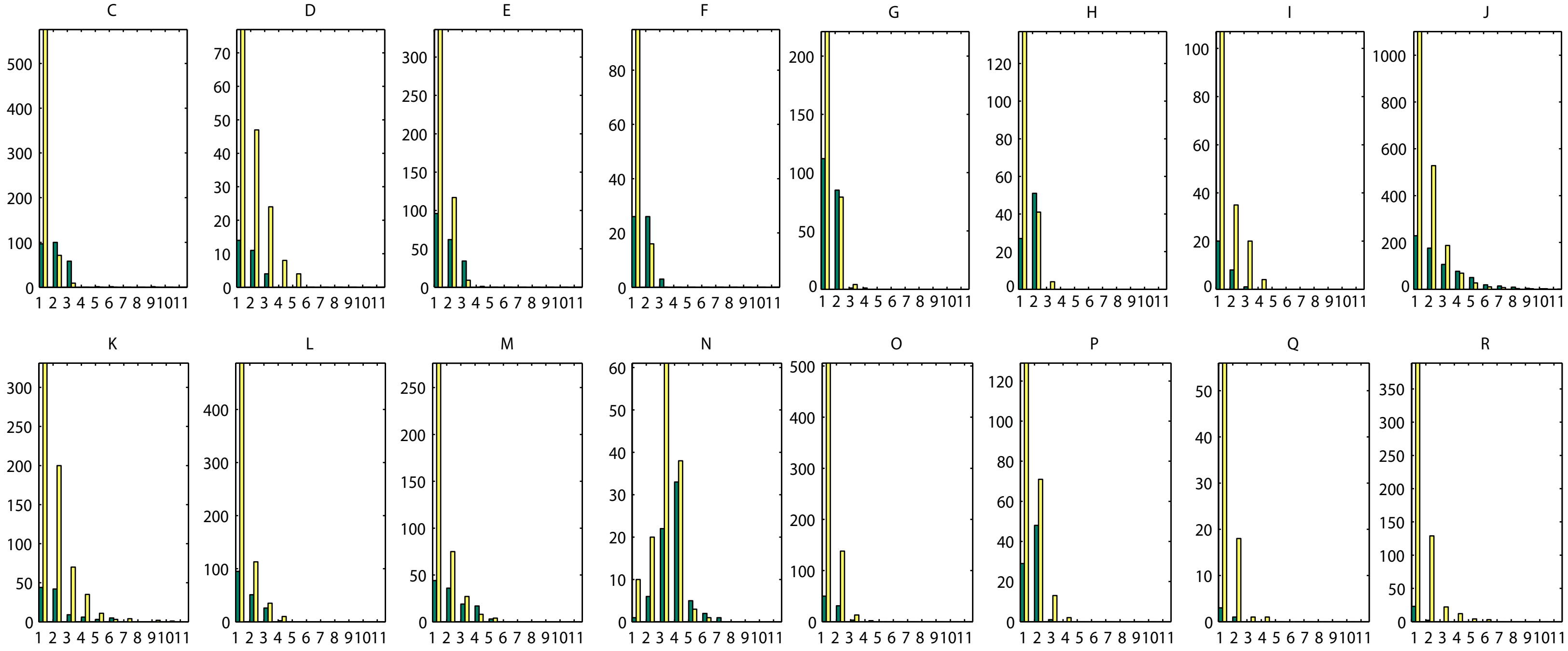

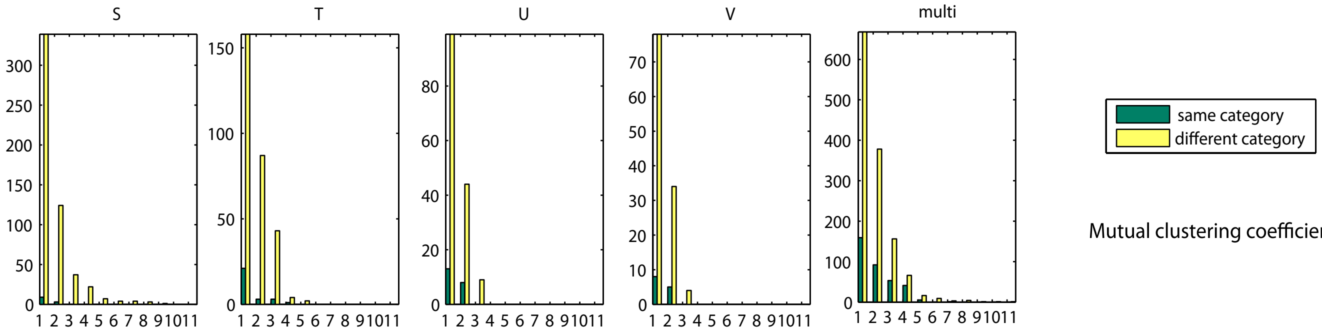

Mutual clustering coefficient

x label	intervals
1	0
2	8.62
3	17.23
4	25.85
5	34.47
6	43.09
7	51.71
8	60.32
9	68.94
10	77.55
11	86.17

Figure S4D

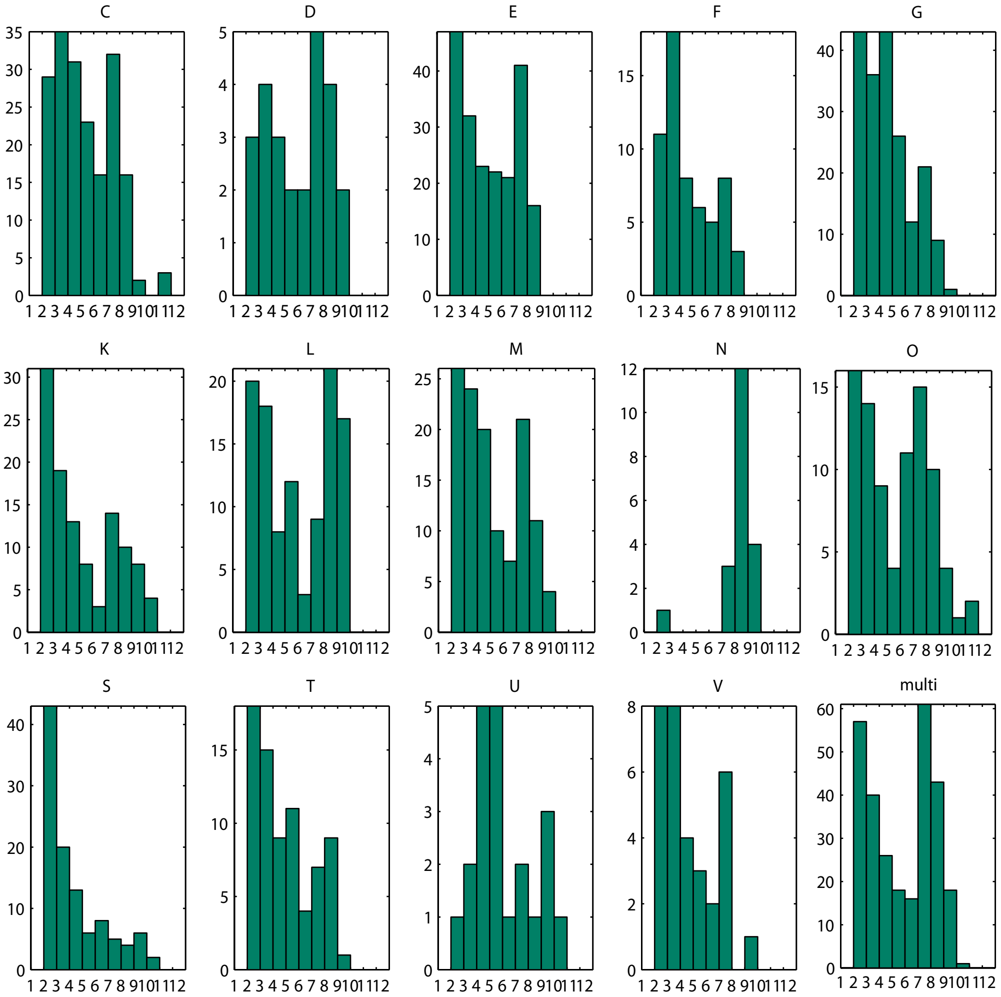

Node degree

x label	intervals
1	0
2	1
3	2
4	3
5	4
6	5
7	6
8	10
9	20
10	50
11	100
12	149

Figure S4E

COG Categories and Number of Proteins in Combined Network

COG	COG Description	Number of proteins
J	Translation, ribosomal structure and biogenesis	133
K	Transcription	110
L	Replication, recombination and repair	108
D	Cell cycle control, cell division, chromosome partitioning	25
V	Defense mechanisms	32
T	Signal transduction mechanisms	74
M	Cell wall/membrane/envelope biogenesis	123
N	Cell motility	20
U	Intracellular trafficking, secretion, and vesicular transport	21
O	Posttranslational modification, protein turnover, chaperones	86
C	Energy production and conversion	187
G	Carbohydrate transport and metabolism	191
E	Amino acid transport and metabolism	202
F	Nucleotide transport and metabolism	59
H	Coenzyme transport and metabolism	86
I	Lipid transport and metabolism	43
P	Inorganic ion transport and metabolism	115
Q	Secondary metabolites biosynthesis, transport and catabolism	22
R	General function prediction only	134
S	Function unknown	110
