# Supplementary material for: The Modular Organization of Protein Interactions in Escherichia coli
Source: PLoS Comput Biol. 2009 Oct 2;5(10):e1000523. doi: 10.1371/journal.pcbi.1000523 (PMC2739439; doi:10.1371/journal.pcbi.1000523)
Supplement: Figure S5 — Topological relationships of COG functional categories within the three derived networks. (A) Number of interactions in the Hu et al. TAP and functional network between each pair of COG categories. Each combination of COG categories is coloured according to the significance (Z-score) of enrichment (red) or depletion (blue) of interactions compared with values obtained from 100 randomly generated networks. (B) Shortest path length between COG categories in the combined, Hu et al. TAP and functional networks. COG category combinations are coloured by the deviation of their shortest path length from the average for the network (red = enrichment, blue = depletion). COG category codes for (A and B) as shown in (C). ‘multi’ = proteins assigned to multiple COG categories. (C) Description of COG functional categories and numbers of proteins in each category associated with each network. Colours were obtained from the COG website (http://www.ncbi.nlm.nih.gov/COG/). (2.33 MB PDF) [file pcbi.1000523.s006.pdf]

(A)

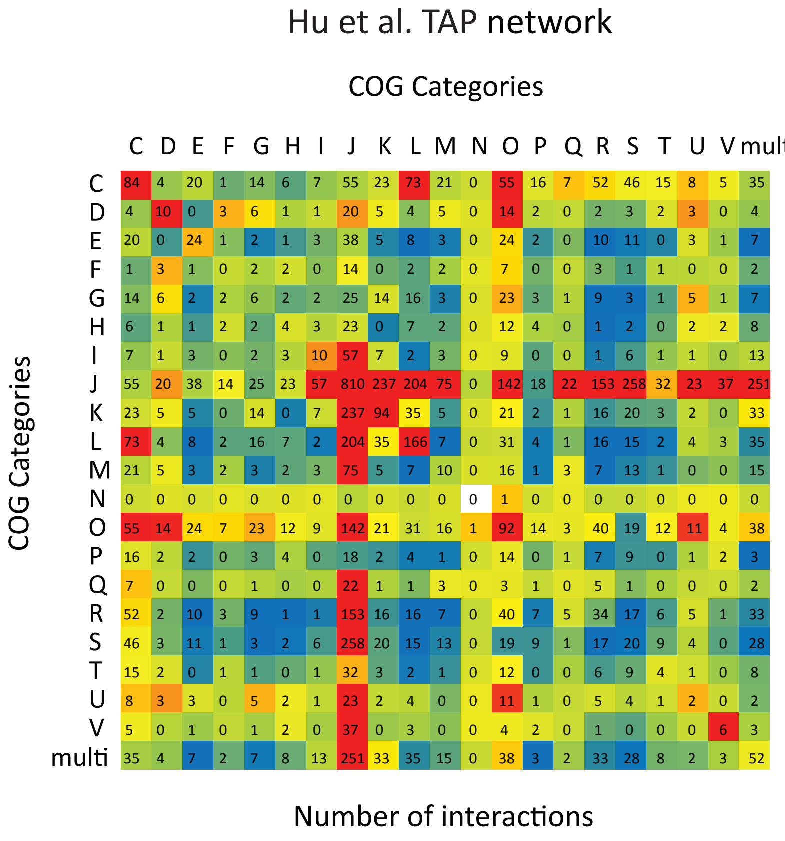

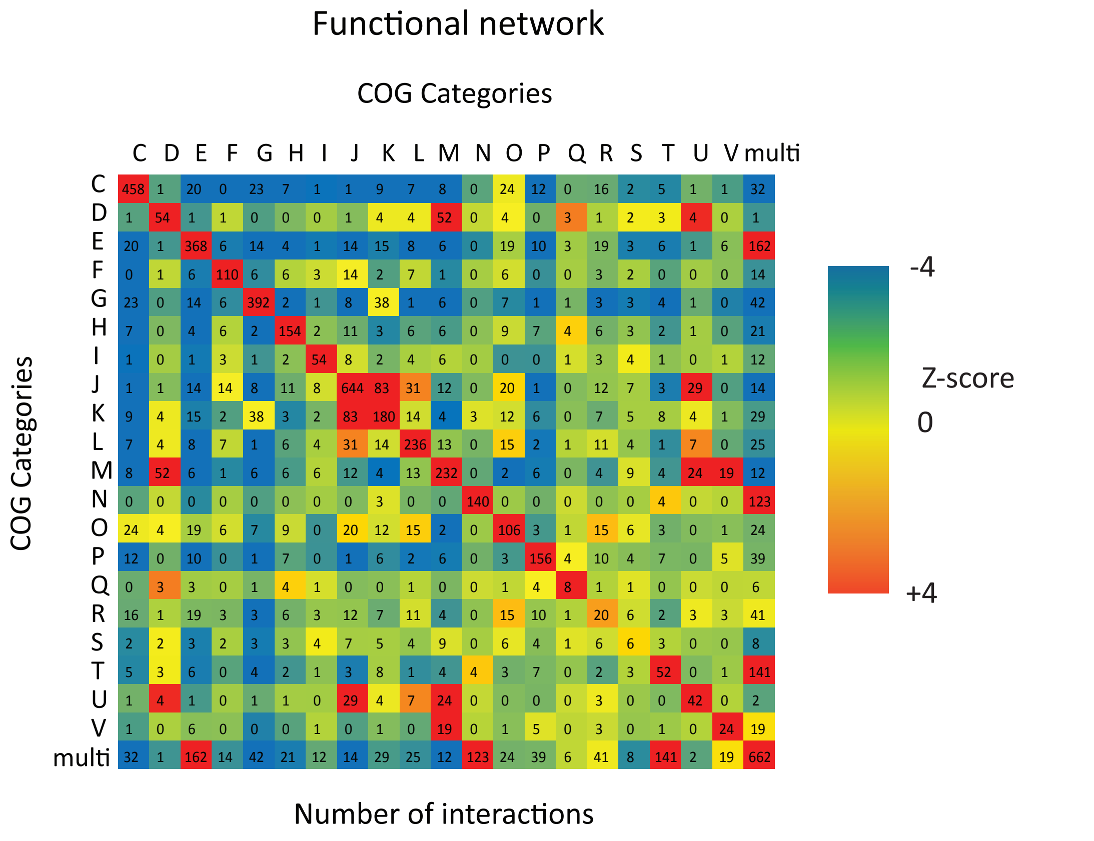

(B)

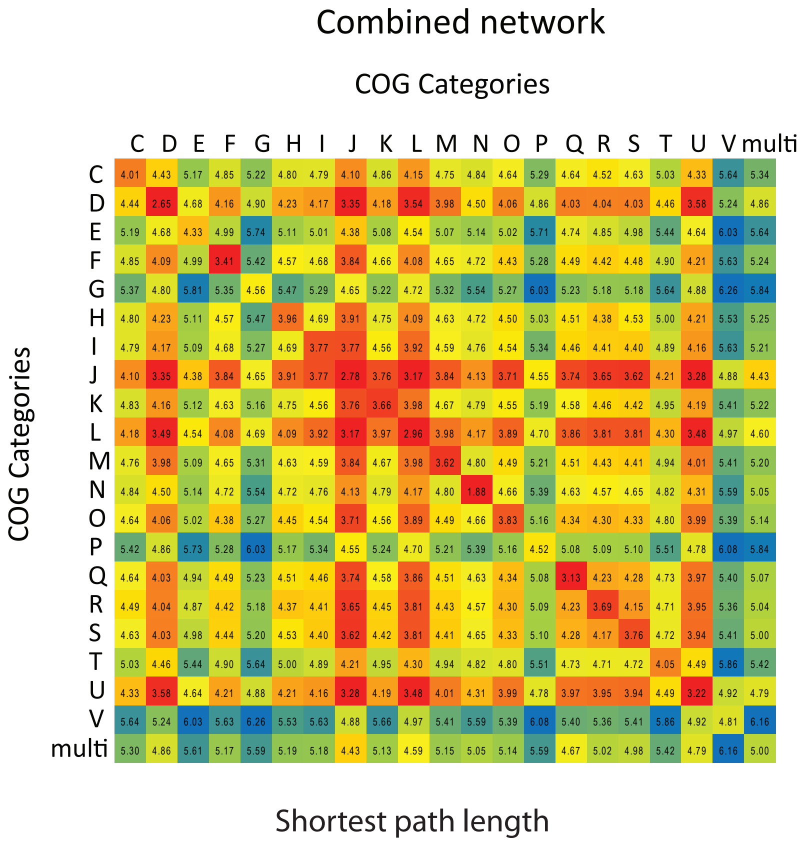

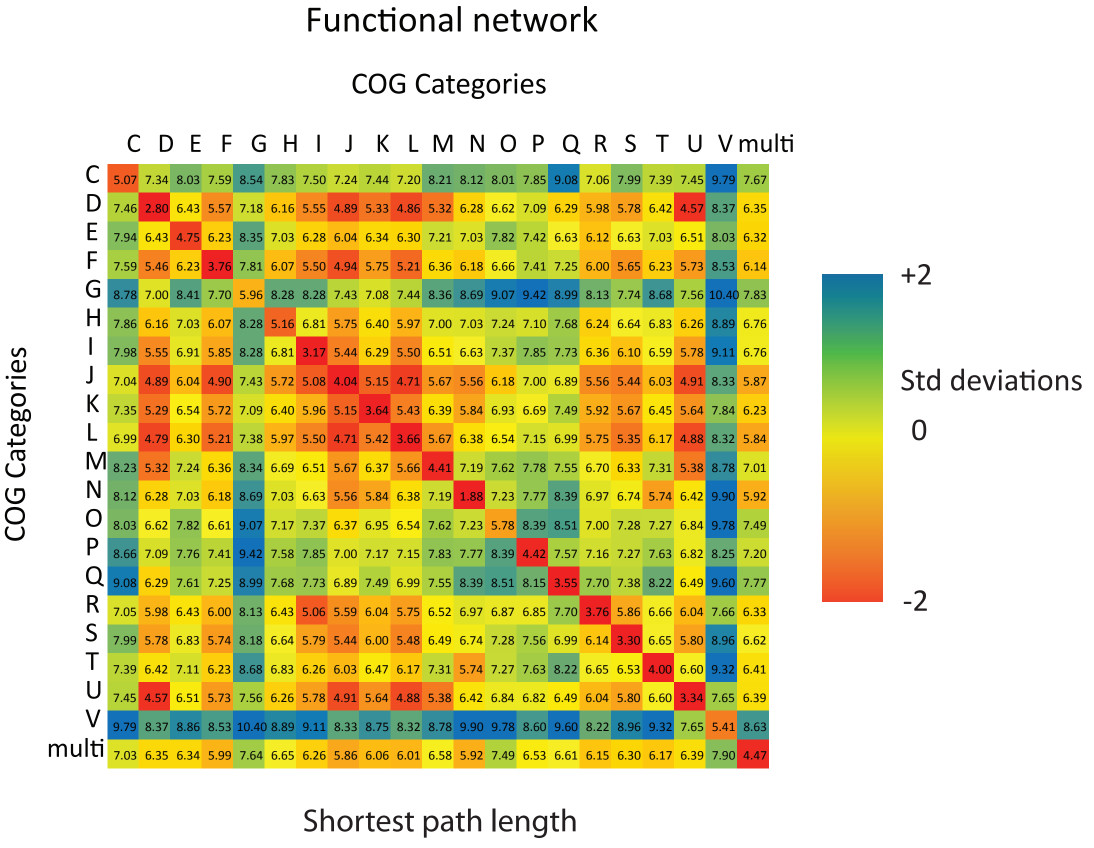

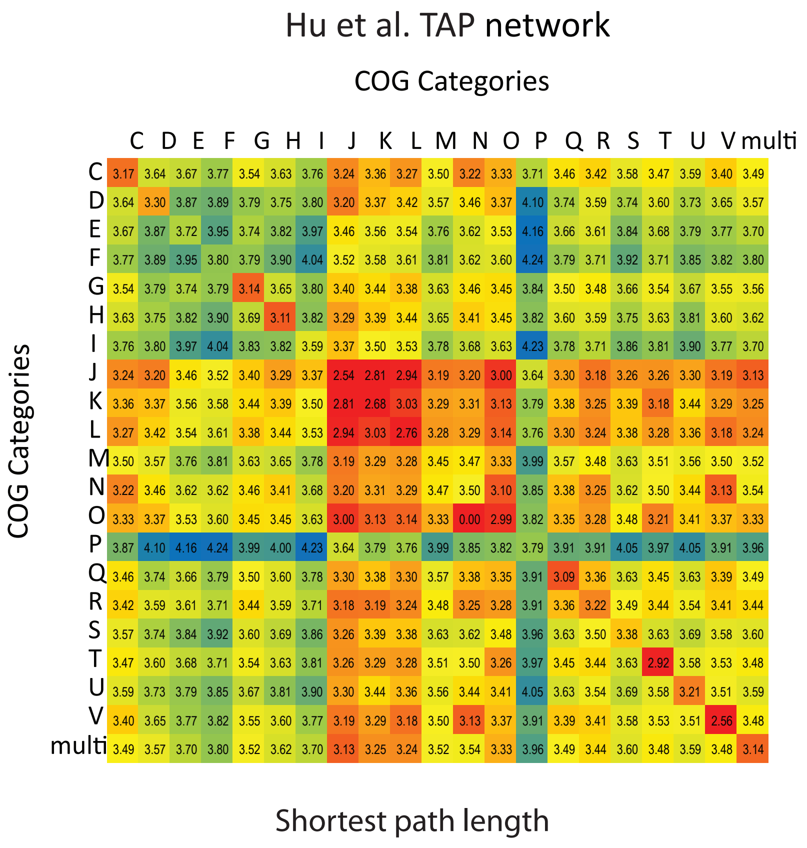

(C)

Number of proteins

COG	COG Description	Combined	Functional	Yu et al. TAP
J	Translation, ribosomal structure and biogenesis	133	118	111
K	Transcription	110	96	45
L	Replication, recombination and repair	108	86	79
D	Cell cycle control, cell division, chromosome partitioning	25	23	13
V	Defense mechanisms	32	30	9
T	Signal transduction mechanisms	74	63	25
M	Cell wall/membrane/envelope biogenesis	123	113	43
N	Cell motility	20	20	1
U	Intracellular trafficking, secretion, and vesicular transport	21	20	9
O	Posttranslational modification, protein turnover, chaperones	86	70	49
C	Energy production and conversion	187	166	63
G	Carbohydrate transport and metabolism	191	178	39
E	Amino acid transport and metabolism	202	186	45
F	Nucleotide transport and metabolism	59	55	13
H	Coenzyme transport and metabolism	86	82	26
I	Lipid transport and metabolism	43	41	23
P	Inorganic ion transport and metabolism	115	102	32
Q	Secondary metabolites biosynthesis, transport and catabolism	22	17	8
R	General function prediction only	134	78	82
S	Function unknown	110	45	79

Figure S5
