# Supplementary material for: The Modular Organization of Protein Interactions in Escherichia coli
Source: PLoS Comput Biol. 2009 Oct 2;5(10):e1000523. doi: 10.1371/journal.pcbi.1000523 (PMC2739439; doi:10.1371/journal.pcbi.1000523)
Supplement: Figure S7 — Networks of predicted functional modules. Each graph indicates a network of predicted modules for the combined, Hu et al. TAP and functional networks. Each pie chart shows the proportion of proteins associated with each COG functional category (see inset for colour key). The size of the pie chart indicates the number of proteins associated with each module. Links between modules indicate interactions between proteins in different module. Note the greater functional heterogeneity associated with the Hu et al. TAP modules compared with the functionally derived modules. (0.99 MB PDF) [file pcbi.1000523.s008.pdf]

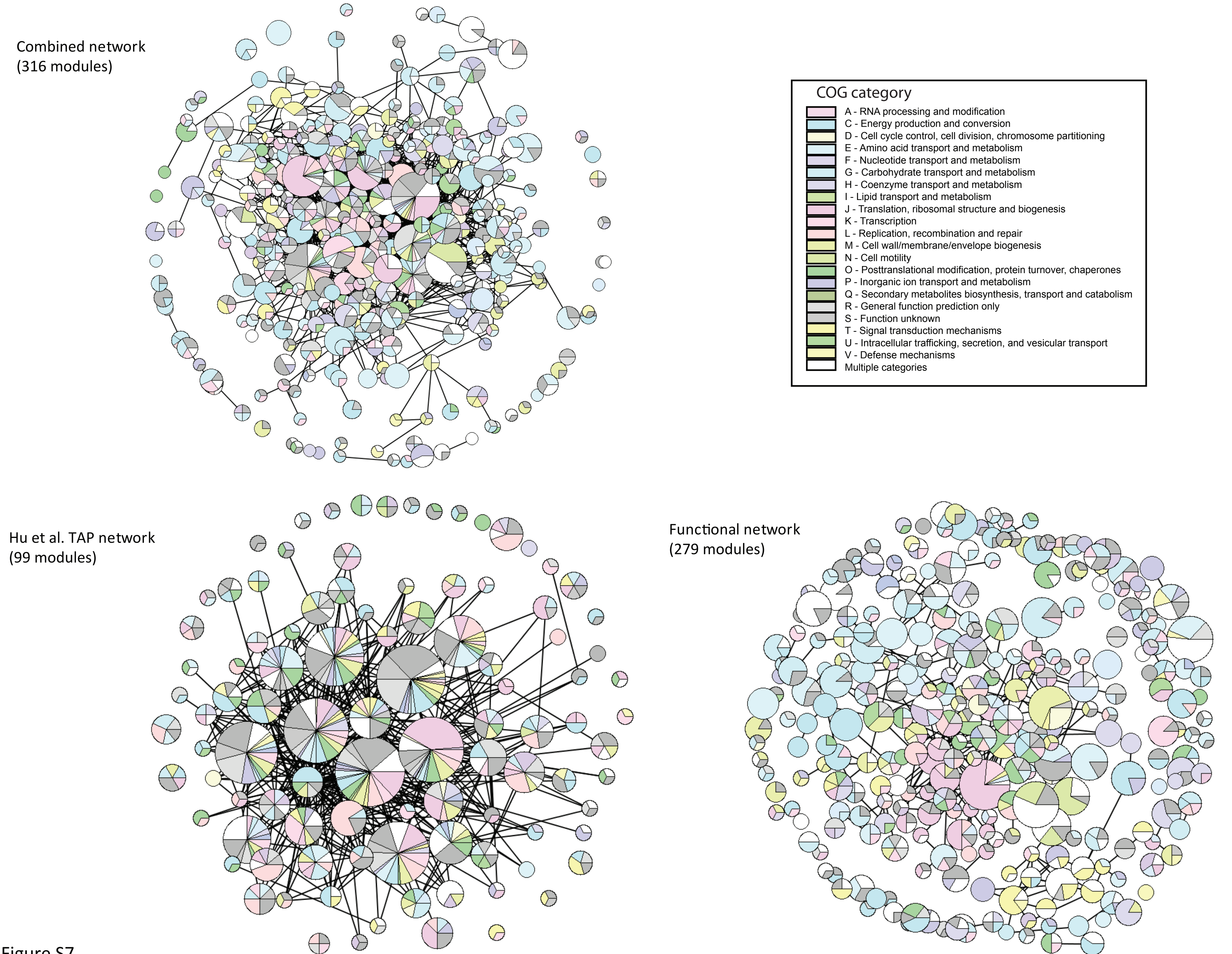
Combined network
(316 modules)
COG category
A - RNA processing and modification
C - Energy production and conversion
D - Cell cycle control, cell division, chromosome partitioning
E - Amino acid transport and metabolism
F - Nucleotide transport and metabolism
G - Carbohydrate transport and metabolism
H - Coenzyme transport and metabolism
I - Lipid transport and metabolism
J - Translation, ribosomal structure and biogenesis
K - Transcription
L - Replication, recombination and repair
M - Cell wall/membrane/envelope biogenesis
N - Cell motility
O - Posttranslational modification, protein turnover, chaperones
P - Inorganic ion transport and metabolism
Q - Secondary metabolites biosynthesis, transport and catabolism
R - General function prediction only
S - Function unknown
T - Signal transduction mechanisms
U - Intracellular trafficking, secretion, and vesicular transport
V - Defense mechanisms
Multiple categories
Hu et al. TAP network
(99 modules)
Functional network
(279 modules)

Figure S7
