# Supplementary material for: The Modular Organization of Protein Interactions in Escherichia coli
Source: PLoS Comput Biol. 2009 Oct 2;5(10):e1000523. doi: 10.1371/journal.pcbi.1000523 (PMC2739439; doi:10.1371/journal.pcbi.1000523)
Supplement: Figure S9 — Network properties associated with gene family membership and laterally transferred genes. (A) Graphs comparing network properties (node degree, betweenness centrality and shortest path length) with gene family membership for the three networks. For the graphs relating betweenness centrality and shortest path lengths for the combined network, also included are the results from 100 ‘random’ networks sharing the same degree distribution as the combined network. The bottom graph indicates the frequency of proteins that interact (according to the combined network) with different numbers of proteins from the same family. Protein families were obtained with reference to the COGENT++ database [33]. (B) Graphs comparing network properties (betweenness centrality and shortest path length) with the origin of a protein (LGT versus non-LGT) for the three networks presented in this study. For each network, LGT genes tend to have lower values of betweenness indicating their peripheral position within the respective networks. The two tailed distribution of shortest path lengths observed for the functional network, highlights the finding that a proportion of LGT genes within this network, occur within discrete interconnected modules. (0.70 MB PDF) [file pcbi.1000523.s010.pdf]

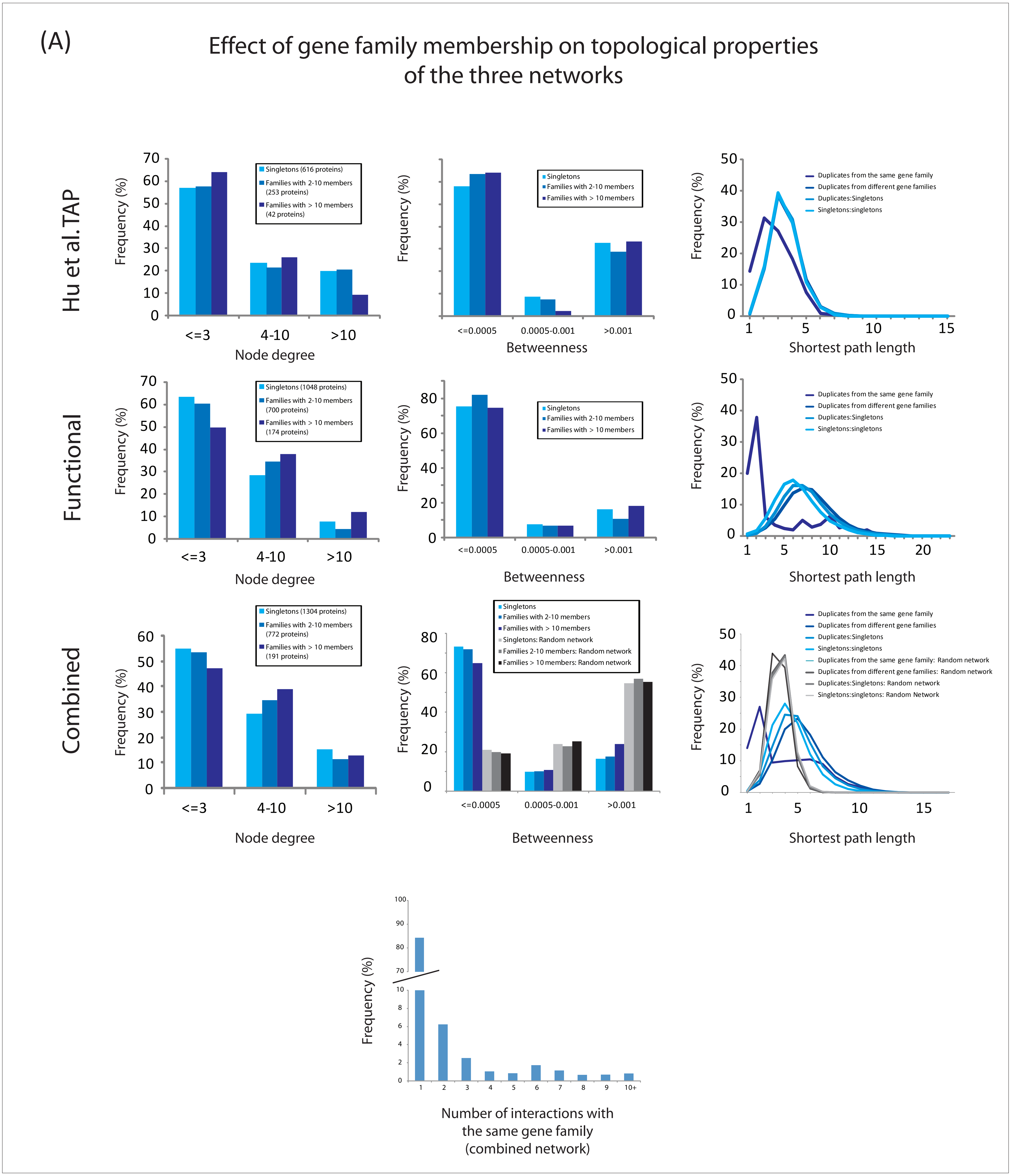

(A)
Effect of gene family membership on topological properties of the three networks
Hu et al. TAP
Functional
Combined
Frequency (%)
Node degree
<=3
4-10
>10
Singletons (616 proteins)
Families with 2-10 members (253 proteins)
Families with > 10 members (42 proteins)
Singletons (1048 proteins)
Families with 2-10 members (700 proteins)
Families with > 10 members (174 proteins)
Singletons (1304 proteins)
Families with 2-10 members (772 proteins)
Families with > 10 members (191 proteins)
Betweenness
<=0.0005
0.0005-0.001
>0.001
Singletons
Families with 2-10 members
Families with > 10 members
Singletons: Random network
Families 2-10 members: Random network
Families > 10 members: Random network
Shortest path length
Duplicates from the same gene family
Duplicates from different gene families
Duplicates:Singletons
Singletons:singletons
Duplicates from the same gene family: Random network
Duplicates from different gene families: Random network
Duplicates:Singletons: Random network
Singletons:singletons: Random Network
Number of interactions with the same gene family (combined network)

Figure S9A

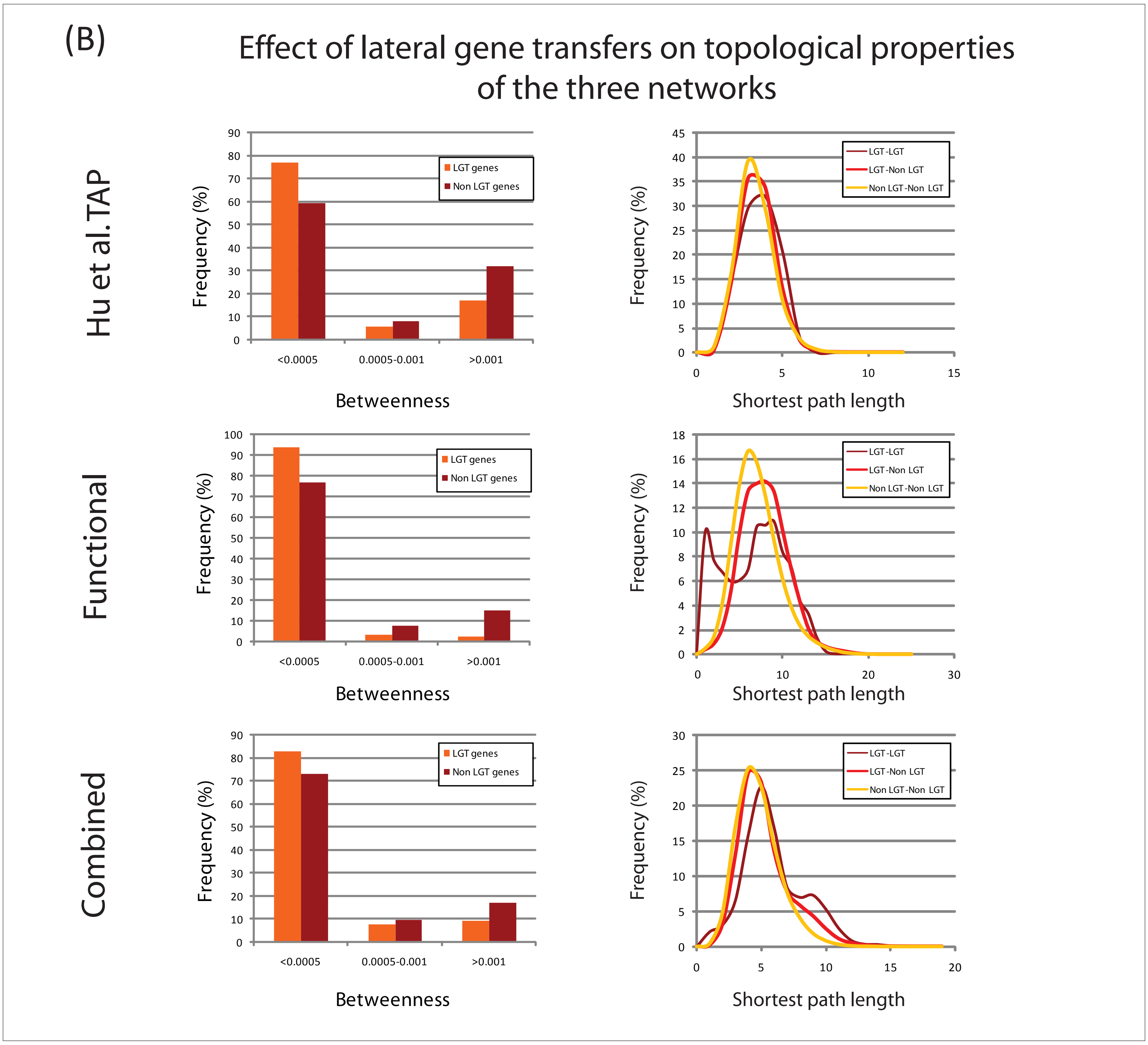
(B)
Effect of lateral gene transfers on topological properties of the three networks
Hu et al. TAP
Functional
Combined
Frequency (%)
Betweenness
Shortest path length
LGT genes
Non LGT genes
LGT-LGT
LGT-Non LGT
Non LGT-Non LGT
<0.0005
0.0005-0.001
>0.001

Figure S9B
